# Associations between biomarkers of inflammation and depressive symptoms—potential differences between diabetes types and symptom clusters of depression

**DOI:** 10.1038/s41398-024-03209-y

**Published:** 2025-01-11

**Authors:** Christian Herder, Anna Zhu, Andreas Schmitt, Maria C. Spagnuolo, Bernhard Kulzer, Michael Roden, Norbert Hermanns, Dominic Ehrmann

**Affiliations:** 1 Institute for Clinical Diabetology, German Diabetes Center (DDZ), Leibniz Center for Diabetes Research at Heinrich Heine University Düsseldorf, Düsseldorf, Germany; 2 German Center for Diabetes Research (DZD), München-Neuherberg, Germany; 3 Department of Endocrinology and Diabetology, Medical Faculty and University Hospital Düsseldorf, Heinrich Heine University Düsseldorf, Düsseldorf, Germany; 4 Research Institute of the Diabetes Academy Mergentheim (FIDAM), Bad Mergentheim, Germany; 5 Diabetes Center Mergentheim (DZM), Bad Mergentheim, Germany; 6 Department of Clinical Psychology and Psychotherapy, University of Bamberg, Bamberg, Germany

**Keywords:** Psychiatric disorders, Biomarkers

## Abstract

Inflammation is a probable biological pathway underlying the relationship between diabetes and depression, but data on differences between diabetes types and symptom clusters of depression are scarce. Therefore, this cross-sectional study aimed to compare associations of a multimarker panel of biomarkers of inflammation with depressive symptoms and its symptom clusters between people with type 1 diabetes (T1D) and type 2 diabetes (T2D). This cross-sectional study combined data from five studies including 1260 participants (*n* = 706 T1D, *n* = 454 T2D). Depressive symptoms were assessed using the Center for Epidemiological Studies-Depression Scale (CES-D). Serum levels of 92 biomarkers of inflammation were quantified with proximity extension assay technology. After quality control, 76 biomarkers of inflammation remained for statistical analysis. Associations between biomarkers and depressive symptom scores and clusters (cognitive-affective, somatic, anhedonia) were estimated with multivariable linear regression models. Nine biomarkers were positively associated with depressive symptoms in the total sample (CCL11/eotaxin, CCL25, CDCP1, FGF-21, IL-8, IL-10RB, IL-18, MMP-10, TNFRSF9; all *p* < 0.05) without interaction by diabetes type. Associations differed for eight biomarkers (*p*_interaction_ < 0.05). TNFβ was inversely associated with depressive symptoms in T1D, whereas three biomarkers (GDNF, IL-18R1, LIF-R) were positively associated with depressive symptoms in T2D. For the remaining four biomarkers (CD6, CD244, FGF-5, IFNγ) associations were not significant in either subgroup. Biomarker associations were more pronounced with somatic and anhedonia than with cognitive-affective symptoms. These results indicate that different proinflammatory pathways may contribute to depression in T1D and T2D and that there may be a symptom specificity in the link between subclinical inflammation and depression.

## Introduction

Depression is one of the most frequent mental comorbidities of type 1 diabetes (T1D) and type 2 diabetes (T2D). The lifetime risk of major depression in the general population has been estimated to reach ~16% [1], but for individuals with diabetes it is probably about twofold higher [2–4]. This excess risk in people with diabetes is important because depression is associated with less optimal diabetes management, higher risk of diabetes-related complications such as cardiovascular disease, nephropathy and cancer, and premature mortality [5–7].

One common mechanism that contributes to the development of diabetes, including its complications and depression as a comorbidity, is subclinical inflammation [8–11]. Both cross-sectional and prospective studies have demonstrated associations of biomarkers of subclinical inflammation with both subclinical depression and clinical depression, i.e. major depressive disorder (MDD), in the general population [12–16]. Additionally, there is evidence that people with subclinical inflammation may be less responsive to antidepressant treatment [6, 17].

Given the higher risk for depression among people with diabetes, data on the potential relevance of inflammation in this particular population group are still relatively scarce [18, 19]. We previously showed that associations between biomarkers of subclinical inflammation and depressive symptoms may be more pronounced in people with T2D than in those with T1D [20, 21]. This finding could reflect differences in the underlying pathophysiology of both diabetes types, but was based on only six biomarkers so more comprehensive studies with better immunological phenotyping are needed.

Another knowledge gap in understanding the bidirectionality between depression and diabetes is the heterogeneity of depression. Depression is a multi-faceted syndrome that includes a variety of different symptoms ranging from cognitive-affective symptoms (e.g., feeling blue, hopelessness), somatic symptoms (e.g., issues with appetite, sleep) and anhedonia (e.g., lack of interest/pleasure). Notably, it is possible for two individuals with a depression diagnosis to share only one symptom based on the 10th revision of the International Statistical Classification of Diseases and Related Health Problems. Thus, identifying possible pathways between depression and inflammation can be masked by potential differential effects of symptom clusters [22]. It has been suggested that subclinical inflammation may be primarily associated with somatic symptoms [23–25], but data are only based on selected biomarkers and few cohorts.

Therefore, this study aimed to characterise the associations between a large panel of biomarkers of inflammation and depressive symptoms in people with diabetes and to test the hypotheses (i) that these associations differ between diabetes types (i.e. stronger in T2D than in T1D) and (ii) that these associations also differ between symptom clusters of depression (i.e. stronger for somatic than for cognitive-affective symptoms).

## Methods

### Study population

This cross-sectional study combines five study samples that underwent standardised phenotyping at a specialised diabetes clinic (Diabetes Center Mergentheim, Bad Mergentheim, Germany). A detailed description of the study populations is given in Supplementary Table 1. In brief, DIAMOS (Diabetes Motivation Strengthening, NCT01009138; [26]), ECCE HOMO (Evaluation of a Stepped Care Approach to Manage Depression in Diabetes, NCT01812291; [27]) and DDCT (Depression and Diabetes Control Trial, NCT02675257) were randomised controlled trials aiming to reduce depressive symptoms and diabetes distress in people with diabetes. DIA-LINK1 and DIA-LINK2 (Towards a Better Understanding of Diabetes Distress, Depression and Poor Glycaemic Control in T1D/T2D, NCT03811132, NCT04438018; [28]) were prospective observational studies using ecological momentary assessment to analyse associations and mediating links between depressive symptoms, diabetes distress and glycaemic outcomes in people with T1D and T2D, respectively. The present study used data and serum samples from the baseline examinations of these five studies. All baseline examinations were conducted before randomisation in the RCTs.

All studies were approved by the relevant ethics boards, i.e. either by the Ethics Committee of the State Medical Chamber of Baden-Württemberg, Germany (file numbers: DIAMOS, 2009-034-f; ECCE-HOMO, F-2013-011; DDCT, F-2015-056) or the Ethics Committee of the German Psychological Society (DIA-LINK1, NH082018; DIA-LINK2: HermannsNorbert2020-03-05AM) and performed in accordance with the Declaration of Helsinki. All participants provided written informed consent.

In total, data from 1226 participants of these studies were available. After exclusion of people with (i) diabetes types other than type 1 or type 2 (*n* = 7), (ii) missing covariates for statistical analysis (*n* = 6), (iii) missing data for biomarkers of subclinical inflammation (mainly due to missing serum samples; *n* = 50) and (iv) missing data on depressive symptoms (*n* = 20), the analysis dataset was based on 1160 people, 706 of whom with type 1 diabetes and 454 with type 2 diabetes (Supplementary Fig. 1)

### Assessment of depressive symptoms

Depressive symptoms were assessed using the German version of the Center for Epidemiological Studies-Depression Scale (CES-D) [29, 30], a sensitive 20-item instrument to detect depressive symptoms within the previous week and to monitor changes over time [31, 32]. The questionnaires were filled in by the study participants with precise instructions and the opportunity to clarify any questions. Each CES-D item is scored from 0 (“rarely or none of the time”) to 3 (“most or almost all the time”) with a summary score ranging from 0 to 60 and higher scores indicating stronger depressive symptoms. We used the continuous CES-D symptom score (rather than a binary, cut-off-based depression variable) in all analyses to make optimal use of the variation in depressive symptoms. Symptom clusters of depressive symptoms were identified according to the recommendations by Shafer [33], i.e. subscores were calculated for cognitive-affective symptoms (items 3, 6, 9, 10, 14, 17, 18), somatic symptoms (items 1, 2, 5, 7, 11, 13, 20) and anhedonia symptoms (items 4, 8, 12, 16 [reversed scoring]).

### Measurement of biomarkers of inflammation

Fasting blood samples were taken between 06:30 a.m. and 08:00 a.m. and serum was stored at -80°C until analysis. Serum levels of biomarkers of inflammation were quantified using the OLINK Target 96 Inflammation assay (Olink, Uppsala, Sweden) as described [34, 35]. This assay is based on proximity extension assay (PEA) technology and designed for the simultaneous measurement of 92 protein biomarkers including cytokines, chemokines, growth factors and factors involved in acute inflammatory and immune responses, angiogenesis, fibrosis and endothelial activation. In this manuscript, “biomarkers of inflammation” refers to all biomarkers from this panel although some of these biomarkers have additional functions in other pathways as well. The PEA provides a relative quantification of protein levels which are given as normalised protein expression (NPX) values. The NPX values are comparable in their distribution to log_2_-transformed biomarker levels.

Supplementary Table 2 lists all biomarkers with UniProt numbers, gene symbols, intra-assay coefficients of variation (CV) and inter-assay CV. Intra- and inter-assay CV were calculated based on three control serum samples measured in duplicates on each plate (*n* = 20) as in previous studies [34, 35]. We had a priori defined threshold levels of intra-assay CV > 15%, inter-assay CV > 20% and a proportion of >25% of values below the respective limit of detection for exclusion of biomarkers from further analysis. Six biomarkers fulfilled all three criteria, and additional ten biomarkers were excluded based on the third criterion so that 76 biomarkers of inflammation remained for statistical analysis.

### Assessment of covariates

Demographic, anthropometric and clinical data were assessed as described before [26–28]. Information on demographic and diabetes-related characteristics comprised age, sex, height and weight (from which BMI was calculated), diabetes type, known diabetes duration, diabetes treatment and co-medication, which was extracted from medical records or interview data. Data about the prevalence or history of diabetes-related complications were based on an entry examination including laboratory analysis and recorded complications in the medical files. History of myocardial infarction, stroke or peripheral arterial occlusive disease was based on previous events or previous revascularization measures. Diabetes-related chronic kidney disease was defined based on a glomerular filtration rate of <60 ml/min/1.73 m² and/or persistent micro-/macroalbuminuria. Diabetic retinopathy was established by an ophthalmologic eye examination or previous laser coagulation treatment. Diabetic neuropathy was based on the neuropathy deficit score.

### Statistical analysis

Baseline characteristics and biomarkers of inflammation are presented as mean (SD) and counts (%) for continuous and categorical variables, respectively. Differences between people with T1D and T2D were tested by using Pearson’s chi-squared test (for categorical variables) or the Wilcoxon rank-sum test (for continuous variables). We additionally compared the baseline characteristics by study cohorts. The correlation among biomarkers of inflammation was analysed by calculating Pearson’s correlation coefficients.

Associations between biomarkers of inflammation (independent variables, separate models for standardised serum levels of each biomarker) and CES-D scores or symptom clusters (dependent variables, also standardised) were estimated using multivariable linear regression models. We reported standardised regression coefficients β (per 1 SD of standardised biomarkers level) and *p*-values from regression models adjusted for different covariates. Model 1 was adjusted for age, sex and study cohort. Model 2 was additionally adjusted for BMI, HbA1c, diabetes duration, total cholesterol, triglycerides, use of lipid-lowering drugs, use of NSAIDs, use of antithrombotic medication and use of antidepressant medication. Model 3 was additionally adjusted for the number of diabetes-related complications. All analyses were further stratified by diabetes type. In addition, differences in the association between biomarkers of inflammation and depressive symptoms were assessed by analysing the interaction between biomarkers of inflammation and diabetes type.

For data visualisation, we plotted the correlation matrix of biomarkers of inflammation and present heat maps to compare biomarkers of inflammation that were significantly associated with CES-D scores and depressive symptom clusters among study participants.

We considered *P*-values of <0.05 to indicate statistically significant differences or associations. Given the exploratory nature of our study using a panel of multiple biomarkers of inflammation and given the complex correlations between these biomarkers we refrained from adjustment for multiple testing. This approach reduces the risk of type II errors and also the overemphasis on *P*-values.

All analyses were conducted in R software (version: 4.2.2, R Core Team, R Foundation for Statistical Computing, Vienna, Austria).

## Results

### Study population

The characteristics of the total study sample and stratified by diabetes type are shown in Table 1. People with T2D were older, more often male, had a higher BMI, higher HbA1c, shorter time since diabetes diagnosis, lower cholesterol and higher triglyceride levels than people with T1D. Additionally, people with T2D more frequently used lipid-lowering, antithrombotic and antidepressant drugs and had a higher number of comorbidities than people with T1D. Supplementary Table 3 gives an overview of the characteristics of the study participants stratified by cohort. Overall, 20.6% of all study participants were first-generation migrants which is similar to the nationwide percentage of 19.2% [36].

**Table 1:** Characteristics of the study population.

Characteristics	Total	T1D	T2D	*P*
*n*		706	454	
Study				
DIAMOS	340 (27.6)	211 (29.9)	109 (24.0)	<0.001
ECCE-HOMO	244 (21.0)	159 (22.5)	85 (18.7)	
DDCT	203 (17.5)	132 (18.7)	71 (15.6)	
DIA-LINK1	204 (17.6)	204 (28.9)	0	
DIA-LINK2	189 (16.3)	0	189 (41.6)	
Diabetes type				
Type 1 diabetes	706 (60.9)	706 (100.0)	/	<0.001
Type 2 diabetes	454 (39.1)	/	454 (100.0)	
Age (years)	44.9 ± 13.9	38.8 ± 13.0	54.3 ± 9.3	<0.001
Sex, females (%)	611 (52.7)	421 (59.6)	190 (41.9)	<0.001
Body mass index (kg/m²)	29.7 ± 7.2	26.1 ± 4.9	35.3 ± 6.6	<0.001
HbA1c (%)	8.9 ± 1.7	8.9 ± 1.7	9.2 ± 1.7	<0.001
HbA1c (mmol/mol)	73.6 ± 18.5	71.4 ± 18.5	77.0 ± 18.0	<0.001
Time since diagnosis of diabetes (years)	14.9 ± 10.2	16.6 ± 11.3	12.2 ± 7.5	<0.001
Total cholesterol (mg/dl)	193.5 ± 49.8	195.9 ± 42.2	189.8 ± 59.5	0.044
Triglycerides (mg/dl)	162.7 ± 149.4	117.2 ± 92.4	233.4 ± 188.7	<0.001
Lipid-lowering drugs (%)	301 (25.9)	89 (12.6)	212 (46.7)	<0.001
NSAIDs (%)	22 (1.9)	13 (1.8)	9 (2.0)	1
Antithrombotic drugs (%)	195 (16.8)	54 (7.6)	141 (31.1)	<0.001
Antidepressant drugs (%)	71 (6.1)	26 (3.7)	45 (9.9)	<0.001
Number of diabetes-related comorbidities	0.9 ± 1.2	0.6 ± 0.9	1.4 ± 1.4	<0.001
Retinopathy (%)	238 (20.5)	144 (20.4)	94 (20.7)	0.958
Diabetes-related chronic kidney disease (%)	106 (9.1)	36 (5.1)	70 (15.4)	<0.001
Polyneuropathy (%)	426 (36.7)	174 (24.6)	252 (55.5)	<0.001
Diabetic foot (%)	55 (4.7)	18 (2.5)	37 (8.1)	<0.001
PAOD (%)	51 (4.4)	14 (2.0)	37 (8.1)	<0.001
Coronary heart disease (%)	91 (7.8)	20 (2.8)	71 (15.6)	<0.001
Myocardial infarction (%)	42 (3.6)	8 (1.1)	34 (7.5)	<0.001
Stroke (%)	27 (2.3)	5 (0.7)	22 (4.8)	<0.001
CES-D	21.9 ± 11.1	21.7 ± 11.1	22.2 ± 11.3	0.400

Mean ± standard deviation (SD) and n (%) were reported for continuous and categorical variables, respectively.

Diabetes-related comorbidities include retinopathy, diabetes-related chronic kidney disease, polyneuropathy, diabetic foot, PAOD, coronary heart disease, myocardial infarction and stroke (max. 8).

*CES-D* Center for Epidemiological Studies-Depression, *DDCT* Depression and Diabetes Control Trial, *DIA-LINK* 1/2 Towards a Better Understanding of Diabetes Distress, Depression and Poor Glycaemic Control in T1D/T2D, *DIAMOS* Diabetes Motivation Strengthening, *ECCE HOMO* Evaluation of a Stepped Care Approach to Manage Depression in Diabetes, *NSAIDs* non-steroidal anti-inflammatory drugs, *PAOD* peripheral arterial occlusive disease, *T1D* type 1 diabetes, *T2D* type 2 diabetes.

Out of 76 biomarkers of inflammation, serum levels differed between diabetes types for 44 biomarkers (10 higher in T1D, 34 higher in T2D, all *P* < 0.05; Supplementary Table 4). Most biomarkers showed positive pairwise correlations with weak or moderate effect sizes (Supplementary Fig. 2). Among the clinical characteristics and covariates, triglyceride levels, age, BMI, diabetes-related comorbidities and diabetes type were most frequently associated with biomarkers of inflammation (Supplementary Fig. 3). Directions and effect sizes of correlations between biomarkers of inflammation and clinical characteristics are summarised in Supplementary Fig. 4.

### Association between biomarkers of inflammation and depressive symptoms (CES-D score)

**Fig. 1:**
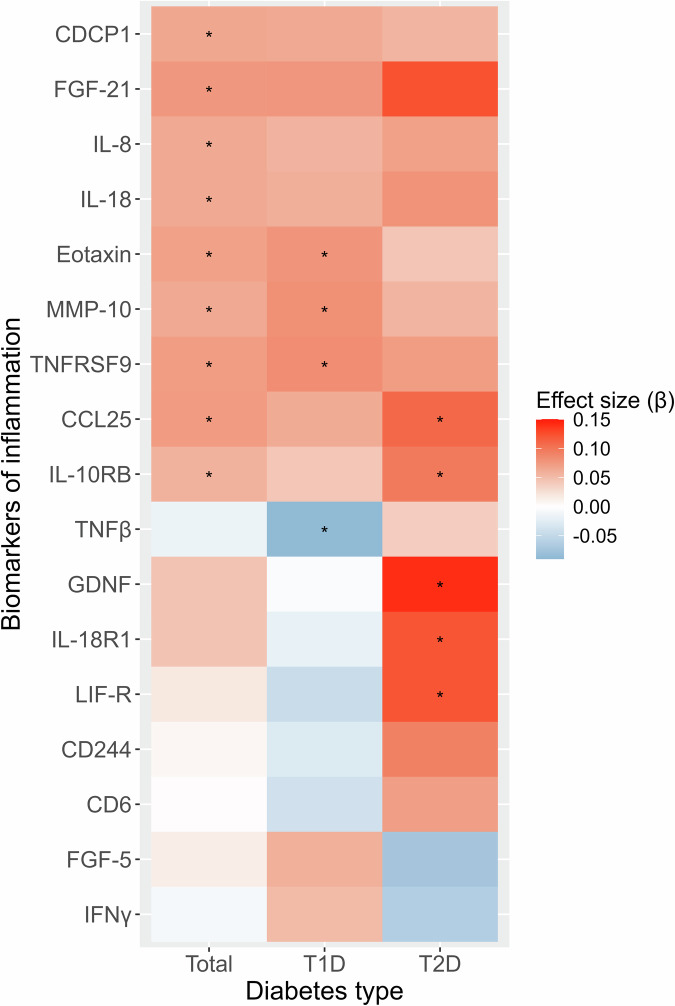
Heat map summarising significant associations of biomarkers of inflammation with the CES-D total score (model 3). *Only results with *P* < 0.05 (in the total sample or at least one of the subgroups) or *p*_interaction_ < 0.05 (CD244, CD6, FGF-5, IFNγ) are shown. β coefficients indicate standardised changes in CES-D scores by per 1-SD increase in biomarkers of inflammation. Regression models were adjusted for age, sex, study, body mass index, HbA1c, diabetes duration, total cholesterol, triglycerides, use of lipid-lowering drugs, non-steroidal anti-inflammatory drugs, antithrombotic medication, antidepressant medication, and number of diabetes-related comorbidities (model 3). T1D, type 1 diabetes; T2D, type 2 diabetes; Total, all people with type 1 or 2 diabetes.

Associations between all biomarkers of inflammation and depressive symptoms in the total study sample and stratified by diabetes type are listed in Supplementary Tables 5-7 for models 1-3, respectively. Results for the biomarkers with significant associations with depressive symptoms in model 3 (fully adjusted) are listed in Table 2 and visualised in Fig. 1. Nine biomarkers of inflammation (CDCP1, FGF-21, IL-8, IL-18, eotaxin, MMP-10, TNFRSF9, CCL25, IL-10RB; see Supplementary Table 2 for full biomarker names) were positively associated with depressive symptoms without significant interaction by diabetes type. Thus, higher levels of these biomarkers of inflammation were associated with greater depressive symptoms (CES-D sum score). In stratified analyses, these associations were also significant for eotaxin, MMP-10 and TNFRSF9 in people with T1D and for CCL25 and IL-10RB in people with T2D.

**Table 2:** Significant associations between biomarkers of inflammation and depressive symptoms (model 3).

Biomarkers	Total		T1D		T2D		*P*_*interaction*_
	β	*P*	β	*P*	β	*P*	
CDCP1	**0.065**	**0.045**	0.063	0.141	0.055	0.275	0.781
FGF-21	**0.078**	**0.021**	0.076	0.062	0.123	0.055	0.413
IL-8	**0.063**	**0.028**	0.056	0.164	0.070	0.099	0.947
IL-18	**0.063**	**0.026**	0.059	0.096	0.078	0.099	0.581
Eotaxin/CCL11	**0.070**	**0.019**	**0.077**	**0.037**	0.043	0.406	0.420
MMP-10	**0.063**	**0.023**	**0.080**	**0.015**	0.055	0.280	0.316
TNFRSF9	**0.072**	**0.011**	**0.083**	**0.033**	0.072	0.089	0.631
CCL25	**0.073**	**0.009**	0.062	0.077	**0.109**	**0.022**	0.761
IL-10RB	**0.057**	**0.046**	0.043	0.238	**0.097**	**0.043**	0.265
TNFβ	−0.015	0.592	**−0.088**	**0.024**	0.037	0.366	**0.011**
GDNF	0.045	0.101	−0.005	0.886	**0.141**	**0.004**	**0.043**
IL-18R1	0.045	0.129	−0.019	0.615	**0.121**	**0.008**	**0.003**
LIF-R	0.018	0.512	−0.045	0.191	**0.121**	**0.010**	**0.012**
CD244	0.006	0.823	−0.028	0.379	0.092	0.069	**0.029**
CD6	0.001	0.967	−0.040	0.242	0.071	0.120	**0.016**
FGF-5	0.014	0.626	0.058	0.088	−0.073	0.147	**0.016**
IFNγ	−0.008	0.762	0.051	0.159	−0.062	0.146	**0.020**

Only results with *p* < 0.05 (in the total sample or at least of the the subgroups) or *p*_interaction_ < 0.05 are listed. See Supplementary Table 7 for a complete list of results.

Regression models were adjusted for age, sex, study, body mass index, HbA1c, diabetes duration, total cholesterol, triglycerides, use of lipid-lowering drugs, non-steroidal anti-inflammatory drugs, antithrombotic medication, antidepressant medication, and number of diabetes-related comorbidities (model 3).

*T1D* type 1 diabetes, *T2D* type 2 diabetes, *Total* people with type 1 or 2 diabetes.

For another eight biomarkers (TNFβ, GDNF, IL-18R1, LIF-R, CD244, CD6, FGF-5, IFNγ), a significant interaction by diabetes type was observed. TNFβ was significantly inversely associated with depressive symptoms in people with T1D only, whereas GDNF, IL18R1 and LIF-R were significantly positively associated with deprerssive symptoms in people with T2D.

### Associations between biomarkers of inflammation and symptom clusters of depression

**Fig. 2:**
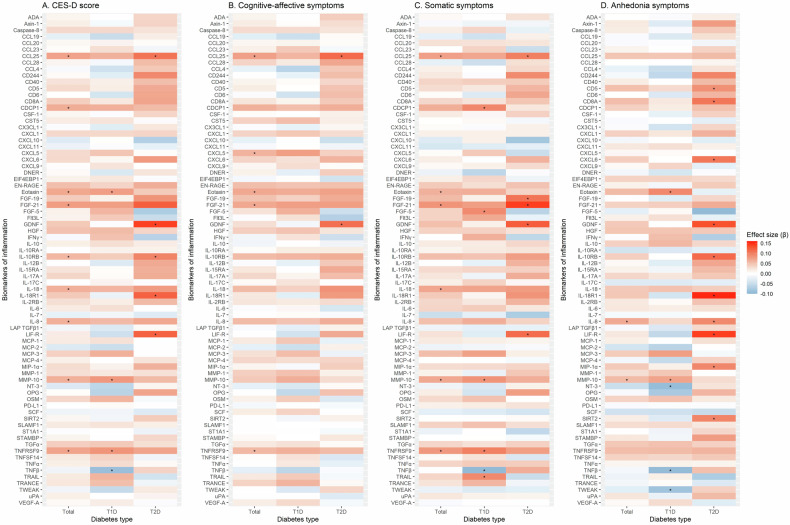
. Heat maps summarising associations of biomarkers of inflammation with the CES-D total score and its symptom clusters. **a** CES-D score. **b** Cognitive-affective symptoms. **c** Somatic symptoms. **d** Anhedoniasymptoms. *P < 0.05 for associations between biomarkers of inflammation and CES-D or symptom clusters. β coefficients indicate standardised changes in CES-D scores or its symptom clusters per 1-SD increase in biomarkers of inflammation. Regression models were adjusted for age, sex, study, body mass index, HbA1c, diabetes duration, total cholesterol, triglycerides, use of lipid-lowering drugs, non-steroidal anti-inflammatory drugs, antithrombotic medication, antidepressant medication, and number of diabetes-related comorbidities (model 3). Abbreviations:T1D, type 1 diabetes; T2D, type 2 diabetes; Total, all people with type 1 or 2 diabetes.

Figure 2A–D summarises all associations of biomarkers of inflammation with the overall depressive symptom (CES-D total) score and the three depressive symptom clusters for the total study sample as well as stratified by diabetes type. For better readability, the four panels are additionally given as separate Supplementary Figs. 5–8. Fully adjusted results for the symptom clusters are also listed in Supplementary Tables 8–10.

Out of the nine biomarkers with positive associations with depressive symptoms in the total sample, four (CCL25, eotaxin, FGF-21, TNFRSF9) were also associated with cognitive-affective symptoms, six (CCL25, FGF-21, eotaxin, IL-18, MMP-10, TNFRSF9) with somatic symptoms and two (IL-8, MMP-10) with anhedonia. In addition, CXCL5 was positively associated with cognitive-affective symptoms in the total study sample.

In the analysis stratified by diabetes type, two biomarkers were positively associated with cognitive-affective symptoms in T2D (CCL25, GDNF). For somatic symptoms, five positive associations (CDCP1, FGF-5, MMP-10, TNFRSF9, TRAIL) and one negative association (TNFβ) were observed in people with T1D, while four biomarkers (FGF-19, FGF-21, GDNF, LIF-R) showed positive associations in people with T2D. For anhedonia, two biomarkers (eotaxin, MMP-10) had positive and three biomarkers (NT-3, TNFβ, TWEAK) had inverse associations in people with T1D, whereas in people with T2D positive associations were found for ten biomarkers (CD5, CD8A, CXCL6, GDNF, IL-10RB, IL-18R1, IL-8, LIF-R, MIP1α, SIRT2).

## Discussion

This study identified novel biomarkers of inflammation that are positively associated with depressive symptoms in people with diabetes. Our data demonstrate that associations between biomarkers of inflammation and depressive symptoms partly differ between T1D and T2D. Additionally, the analysis of symptom clusters supports the hypothesis that positive associations between biomarkers of inflammation are particularly pronounced for somatic symptoms in the total study sample and with anhedonia symptoms in people with T2D.

### Association between biomarkers of inflammation and depressive symptoms (CES-D score) in the total study sample

This study demonstrates positive associations of CDCP-1, FGF-21, IL-8, IL-18, eotaxin/CCL11, MMP-10, TNFRSF9, CCL25 and IL-10RB with depressive symptoms in people with diabetes without interaction with diabetes type. We are not aware of any previous study that assessed the aforementioned biomarkers in people with diabetes except IL-18 as discussed below [20], which underlines the novelty of our findings.

When comparing our results with studies that did not focus on people with diabetes, our data are in line with positive associations of the chemokine IL-8/CXCL8 and the proinflammatory cytokine IL-18 with depression that were found in previous meta-analyses based on study cohorts with a wide range of participant characteristics such as age, BMI and burden of comorbidities [13, 37]. However, no significant association was found for eotaxin/CCL11 [37].

Two small studies on FGF-21 and MDD reported conflicting results. One study found higher FGF-21 levels in people with chronic, early-onset MDD than in healthy people [38], which is in line with our data. Another study reported lower FGF-21 levels in people with MDD compared to healthy people but no associations between FGF-21 and symptom score [39]. There is evidence from mouse models that endogenous FGF-21 counteracts depression-like behaviour [40] so that higher FGF-21 levels might reflect a compensatory response but these data are difficult to extrapolate to depression in humans.

One cross-sectional study in women with and without postpartum depressive symptoms used the same multimarker panel as our analysis [41]. However, there was no overlap in biomarkers showing significant associations with depressive symptoms, which may be attributable to considerable differences in the aetiology of postpartum depression and the development of depression in adulthood that is not related to pregnancy.

Taken together, IL-8/CXCL8 and IL-18 appear related with depressive symptoms irrespectively of diabetes status. Whether the novel associations of the other biomarkers discussed here are specific to people with diabetes is unclear because of the lack of comparable data from other cohorts. Thus, our findings should be corroborated in both cohorts based on people with diabetes and in cohorts from the general population.

### Differences in associations between biomarkers of inflammation and depressive symptoms between T1D and T2D

Given the differences in the aetiology of T1D and T2D in particular regarding the role of inflammation and the immune system, analyses of potential differences between diabetes types are relevant for all comorbidities of diabetes. This study extends the current knowledge about potential differences between diabetes types [20] and provides novel evidence that associations between biomarkers and inflammation and depressive symptoms partly differ between T1D and T2D. Associations for eight biomarkers showed a significant interaction by diabetes type, although only four of them were also significantly associated with depressive symptoms in one of the subgroups.

Higher levels of TNFβ, also known as lymphotoxin-α or TNFSF1, were associated with less pronounced depressive symptoms in people with T1D. In contrast, higher levels of GDNF, IL-18R1 and LIF-R were associated with more pronounced depressive symptoms in people with T2D. Of these four biomarkers, only GDNF has been investigated in the context of depression before. GDNF is a potent neurotrophic factor that promotes the growth and survival of various types of neurons and protects them from oxidative stress. It has been hypothesised that GDNF has anti-depressant properties [42] so that the positive association between GDNF levels and depressive symptoms in T2D in our study could reflect a counterregulation. However, reports on associations with MDD in other studies were conflicting. No differences in GDNF levels between people without and with MDD were found in a recent meta-analysis but the reasons for the large heterogeneity between the studies are not known [43]. Our finding for IL-18R1 supports the aforementioned association of IL-18 with depression and highlights the potential relevance of inflammasome activation and IL-18/IL-18R1 signalling.

This analysis focused on the comparison between T1D and T2D, whereas future studies addressing the heterogeneity of diabetes and depression should also take into account the novel five subtypes as suggested by Ahlqvist et al. [44]. These identified diabetes subtypes differ in circulating biomarkers of inflammation [35] and quality of health [45] but data for depression in the subtypes have not been published yet.

### Associations between biomarkers of inflammation and symptom clusters of depression

Not only diabetes but also depression is a heterogeneous disease with diverse clinical manifestations [46, 47], thus studies focusing on symptom clusters of depression could provide additional insights into the neurobiology of the different facets of depression. In our analysis of biomarkers of inflammation and symptoms clusters, the largest number of significant associations in the total sample were found for somatic symptoms, whereas the stratified analysis showed most associations with anhedonia in people with T2D.

We are not aware of comparable analyses in people with diabetes so that our data extend the current literature on inflammation in the context of the heterogeneity of both diabetes and depression. Previous studies on symptom clusters of depression focused on high-sensitivity C-reactive protein (hsCRP) and few additional biomarkers [46, 47], whereas studies with more comprehensive immunological phenotyping are lacking. In these studies, hsCRP showed the strongest associations with somatic symptoms of depression [23, 25, 46–48]. This observation is supported by multiple lines of evidence linking inflammation with coordinated behavioural changes (termed sickness behaviour) which are related to physical illness and infection [8]. Associations were also found for anhedonia and were weakest with cognitive-affective symptoms [23, 46–48]. This is in line with mechanistic and translational studies that demonstrated the involvement of inflammatory stimuli for the development of anhedonia [49]. Our data are consistent with these aforementioned studies in that we found more evidence for associations of inflammation with somatic symptoms and anhedonia than with cognitive-affective symptoms.

Of note, biomarkers associated with somatic symptoms overlapped largely with biomarkers also associated with the CES-D score overall. In addition, FGF-5, TRAIL and FGF-19 showed positive associations with somatic symptoms in the stratified analysis. So far, only higher levels of the proinflammatory cytokine TRAIL have been implicated with MDD in a small study [50].

In contrast, associations with anhedonia were mainly seen for T2D in the stratified analysis. In addition to biomarkers also associated with the CES-D score in the total study sample, there were positive associations of CD5, CD8A, CXCL6, MIP-1α/CCL3 and SIRT2 with anhedonia. CD5 and CD8A are expressed on T cells which have been implicated in the pathogenesis of depression [51]. CXCL6 and MIP1α/CCL3 add to the growing list of chemokines associated with depression, and a previous meta-analysis supports our finding regarding MIP1α/CCL3 and anhedonia [37]. SIRT2 (sirtuin 2), an NAD-dependent deacetylase, has been linked to various neuroinflammatory diseases but the underlying mechanisms are still poorly understood [52]. On the one hand, SIRT2 has anti-inflammatory properties [52]. On the other hand, SIRT2 inhibitors ameliorate depression-like behaviour in mouse models [52, 53], and SIRT2 expression is also downregulated by anti-depressants [54]. Therefore, the positive association between SIRT2 levels and anhedonia from our study appears biologically plausible.

Unfortunately, there were other aspects of the heterogeneity of depression that we could not address in our study. We did not have data on the temporal relationship between diabetes diagnosis and depressive symptomology. Additionally, we relied on the CES-D score but did not have clinical interviews, data on remission status or, for those with high CES-D scores, information on first or recurrent depression. The proportion of people on antidepressant medication was too small for subgroup analyses but the use of this medication was adjusted for as confounder in our regression models.

### Clinical relevance and implications for precision medicine

Our study identified multiple biomarkers of inflammation that are associated with depressive symptoms in people with diabetes. These include proinflammatory cytokines and their receptors (IL-18, IL-18R1, LIF-R), chemokines (IL-8/CXCL8, eotaxin/CCL11, CCL25, CXCL6, MIP-1α/CCL3), biomarkers involved in the cross-talk between innate and adaptive immunity (TNFRSF9, CD5, CD8A) and lastly biomarkers that might be upregulated in depression as part of a counterregulatory response (FGF-21, GDNF). Since these biomarkers were measured in serum, it is not possible to directly link our findings to specific cell types that might be responsible for their expression and release into the circulation. However, our findings suggest the implication of cell types from both innate and adaptive immunity and point towards a role of cell-cell communication and migration in the inflammation-related processes that are associated with higher depressive symptoms. This is supported by a recent meta-analysis showing that blood counts of multiple subsets of immune cells are altered in people with depression [55].

In order to translate our findings to improved therapy, a more comprehensive phenotyping and the search for distinct upstream regulators that could represent actionable targets would be of interest. Several meta-analyses provided evidence that drugs targeting cytokines such as IL-6 or IL-12/IL-23 reduced depressive symptoms [56]. This suggests that anti-inflammatory drugs could be useful as adjunctive therapy in people with depression. However, most of these data were based on people with depression and inflammatory diseases such as rheumatoid arthritis or systemic lupus erythematosus who are characterised by a more pronounced immune activation than people with depression but without such diseases. Therefore, the potential of anti-inflammatory approaches in the treatment of depression as primary disease remains unclear. Of note, our findings support the hypothesis that different inflammatory mechanisms may be relevant in T1D and T2D and also for specific symptoms of depression. However, the identification of inflammation-related subtypes is still an unmet clinical need in both diabetology and psychiatry but might have considerable potential for patient stratification and implementation of precision medicine [6].

Given the fact that people with subclinical inflammation are also characterised by higher risk of cardiovascular and neurodegenerative diseases, cancer and frailty [9], it remains to be seen to what extent immunomodulatory aproaches might have broader benefits, in particular in the prevention of multimorbidity.

### Strengths and limitations

Strengths of our study include the relatively large sample size, the extensive biomarker measurement, the comprehensive adjustment for confounders and the analyses focusing on diabetes type and on symptom clusters of depression. This study also has limitations. First, the cross-sectional study does not allow conclusions on temporal and causal relationships between inflammation and depressive symptoms. The lack of temporal ordering also precluded us from performing mediation analysis [57] which would be of interest to better understand the bidirectional relationship of diabetes and depression. Second, the study sample consisted of mainly middle-aged people with moderate to high levels of depressive symptoms up to mild/moderate MDD (while people with severe MDD were excluded from the studies for ethical reasons), so our findings might not be generalisable to other age groups or to people with severe forms of MDD. In addition, the study was performed in a tertiary care setting which is characterised by worse glycaemic control compared to all people with diabetes, which might lead to some selection bias. Unfortunately, we did not have data on ethnicity but with respect to the percentage of first-generation migrants our study sample was representative for Germany. Third, the high prevalence of T1D in our study means that our results for the total study sample cannot be directly compared to population-based studies on diabetes which are highly dominated by T2D. Fourth, given the exploratory nature of our study and the complex correlations between biomarkers of inflammation we did not adjust for multiple testing so our results need replication in other studies. Lastly, the biological interpretation of some of the findings is unclear because some of the biomarkers discussed above represent circulating forms of primarily cytoplasmatic or transmembrane proteins, and it is not known how their circulating concentrations in blood are regulated.

## Conclusions

In summary, this study found multiple associations between biomarkers of inflammation and depressive symptoms in people with diabetes and provides evidence that these associations and relevant biomarkers differ depending on diabetes type and symptom clusters of depression. Further elucidation of the specificity for subtypes of diabetes and/or depression could be promising for future patient stratification and the development of more targeted treatment options for depression in diabetes.

## Supplementary information

Supplemental tables and figures

## Data Availability

The data are subject to national data protection laws. Therefore, data cannot be made freely available in a public repository. However, data can be requested through an individual project agreement with FIDAM and DDZ.
