# Supplementary material for: Associations between biomarkers of inflammation and depressive symptoms—potential differences between diabetes types and symptom clusters of depression
Source: Transl Psychiatry. 2025 Jan 11;15:9. doi: 10.1038/s41398-024-03209-y (PMC11724873; doi:10.1038/s41398-024-03209-y)

Supplementary Table 1. Study populations.

Study cohort (sample size)	Study aim	Inclusion criteria	Exclusion criteria	Registration number (ClinicalTrials.gov)	Reference
DIAMOS (Diabetes Motivation Strengthening) (n=340)	RCT aiming at the reduction of diabetes distress using cognitive behavioural interventions	-Diabetes mellitus<br>-Age 18-70 years<br>-Elevated depressive symptoms (CES-D ≥16)<br>-Sufficient German language skills	-Inability to consent<br>-Major depression, schizophrenia/psychotic disorder, severe eating disorder, bipolar disorder, addictive disorder or personality disorder, current use of antidepressant medication or ongoing psychotherapy, being bedridden, or being under guardianship	NCT01009138	(Hermanns 2015)
ECCE-HOMO (Evaluation of a Stepped Care Approach to Manage Depression in Diabetes) (n=260)	RCT examining the efficacy of a stepped care approach for depressed people with diabetes	-Diabetes mellitus<br>-Age 18-70 years<br>-Elevated depressive symptoms (CES-D ≥16) and/or elevated diabetes-related distress (PAID ≥40)<br>-Sufficient German language skills	-Inability to consent<br>-Severe clinical depression according, suicidal ideation, severe somatic illness (e.g. end-stage diabetes complication, terminal stage cancer) or dementia, current psychotherapeutic/psychiatric treatment, current antidepressive medication, schizophrenia/psychotic disorder, severe eating disorder, bipolar disorder, addictive disorder, personality disorder, being bedridden, or being under guardianship	NCT01812291	(Schmitt 2022)
DDCT (Depression and Diabetes Control Trial) (n=213)	RCT evaluating a cognitive-behavioural intervention for people with	-Diabetes mellitus (T1D or T2D)<br>-Diabetes duration ≥1 year<br>-Age 18-70 years<br>-HbA1c >7.5%	-Inability to consent<br>-Severe major depressive disorder, current psychiatric and/or psychotherapeutic treatment, current antidepressive medical	NCT02675257	-

	diabetes, suboptimal glycaemic control and comorbid depressive symptoms and/or diabetes distress	-Elevated depressive symptoms (CES-D ≥16) and/or elevated diabetes distress (PAID ≥ 40)<br>-Sufficient German language skills	treatment, suicidal ideation. acute mental disorder (schizophrenia or other psychotic disorder, bipolar disorder, severe eating disorder, substance use disorder), history of personality disorder, severe somatic illnesses (dialysis-dependent nephropathy, acute cancer, severe heart disease, severe neurologic illness, severe autoimmune disease, terminal illness, bedriddenness, guardianship		
DIA-LINK1 (Towards a Better Understanding of Diabetes Distress, Depression and Poor Glycaemic Control in T1D) (n=208)	Prospective observational study analysing longitudinal associations and mediating links between diabetes distress, depressive symptoms and glycaemic outcomes in people with T1D	-T1D<br>-Diabetes duration ≥1 year<br>-Age 18–70 years<br>-Sufficient German language skills<br>-Compatible smartphone<br><br>Elevated diabetes distress was determined with use of PAID with a cutoff score of ≥40. Elevated depressive symptoms were determined with use CES-D scale with a cutoff score of ≥22.<br><br>Four groups with approx. 50 participants each were established, including those with (i) neither elevated diabetes distress nor depressive symptoms, (ii) elevated diabetes distress	-Inability to consent<br>-Significant cognitive impairment, severe somatic illness or mental disorder, terminal illness, or being bedridden	NCT03811132	(Ehrmann 2022)

		but no elevated depressive symptoms, (iii) no elevated diabetes distress but elevated depressive symptoms, and (iv) elevated diabetes distress and elevated depressive symptoms.			
DIA-LINK2 (Towards a Better Understanding of Diabetes Distress, Depression and Poor Glycaemic Control in T2D) (n=205)	Prospective observational study analysing longitudinal associations and mediating links between diabetes distress, depressive symptoms and glycaemic outcomes in people with T2D	-T2D<br>-Diabetes duration ≥1 year<br>-Age 18–70 years<br>-Sufficient German language skills<br>-Compatible smartphone<br><br>Elevated diabetes distress was determined with use of PAID with a cutoff score of ≥40. Elevated depressive symptoms were determined with use CES-D scale with a cutoff score of ≥22.<br><br>Four groups with approx. 50 participants each were established, including those with (i) neither elevated diabetes distress nor depressive symptoms, (ii) elevated diabetes distress but no elevated depressive symptoms, (iii) no elevated diabetes distress but elevated depressive symptoms, and (iv) elevated	-Inability to consent<br>-Significant cognitive impairment, severe somatic illness or mental disorder, terminal illness, or being bedridden	NCT04438018	-

		diabetes distress and elevated depressive symptoms.			

CESD, Center for Epidemiological Studies-Depression; PAID, Problem Areas In Diabetes; RCT, randomised controlled trial; T1D, type 1 diabetes; T2D, type 2 diabetes.

References:

Ehrmann D, Schmitt A, Priesterroth L, Kulzer B, Haak T, Hermanns N. Time With Diabetes Distress and Glycemia-Specific Distress: New Patient-Reported Outcome Measures for the Psychosocial Burden of Diabetes Using Ecological Momentary Assessment in an Observational Study. Diabetes Care. 2022 Jul 7;45(7):1522-1531. doi: 10.2337/dc21-2339.

Hermanns N, Schmitt A, Gahr A, Herder C, Nowotny B, Roden M, et al. The effect of a Diabetes-Specific Cognitive Behavioral Treatment Program (DIAMOS) for patients with diabetes and subclinical depression: results of a randomized controlled trial. Diabetes Care. 2015 Apr;38(4):551-60. doi: 10.2337/dc14-1416.

Schmitt A, Kulzer B, Reimer A, Herder C, Roden M, Haak T, et al. Evaluation of a Stepped Care Approach to Manage Depression and Diabetes Distress in Patients with Type 1 Diabetes and Type 2 Diabetes: Results of a Randomized Controlled Trial (ECCE HOMO Study). Psychother Psychosom. 2022;91(2):107-122. doi: 10.1159/000520319.

Supplementary Table 2. Biomarkers in the OLINK Inflammation panel and assay characteristics

Biomarker	Full name	UniProt No	Gene symbol	Intra-assay CV (%)	Inter-assay CV (%)	LOD (NPX)	Percentage of samples below LOD
ADA	Adenosine deaminase	P00813	ADA	1.6	5.2	1.72	0
ARTN*	Artemin	Q5T4W7	ARTN	57.5	43.3	0.70	55.5
AXIN1	Axis inhibition protein 1	O15169	AXIN1	4.1	9.0	1.47	1.6
Beta-NGF*	Beta-nerve growth factor	P01138	NGF	4.5	4.0	1.42	97.8
CASP-8	Caspase-8	Q14790	CASP8	2.4	11.7	2.15	0.5
CCL3	C-C motif chemokine 3	P10147	CCL3	1.2	2.8	2.08	0
CCL4	C-C motif chemokine 4	P13236	CCL4	1.1	2.3	2.44	0
CCL19	C-C motif chemokine 19	Q99731	CCL19	1.2	2.6	0.80	0
CCL20	C-C motif chemokine 20	P78556	CCL20	1.4	3.6	2.42	0
CCL23	C-C motif chemokine 23	P55773	CCL23	0.9	1.7	1.47	0
CCL25	C-C motif chemokine 25	O15444	CCL25	1.2	2.5	1.67	0
CCL28	C-C motif chemokine 28	Q9NRJ3	CCL28	5.3	8.0	1.30	0
CD5	T-cell surface glycoprotein CD5	P06127	CD5	1.3	2.0	1.52	0
CD6	T-cell differentiation antigen CD6	P30203	CD6	2.1	3.1	0.96	0
CD8A	T-cell surface glycoprotein CD8 alpha chain	P01732	CD8A	1.1	2.0	1.51	0
CD40	Tumor necrosis factor receptor superfamily member 5	P25942	CD40	0.7	1.4	1.62	0
CD244	Natural killer cell receptor 2B4	Q9BZW8	CD244	1.6	2.8	1.02	0
CDCP1	CUB domain-containing protein 1	Q9H5V8	CDCP1	5.6	11.8	-0.29	0
CSF-1	Macrophage colony-stimulating factor 1	P09603	CSF1	0.9	1.1	1.60	0
CST5	Cystatin-D	P28325	CST5	1.5	2.8	0.15	0
CX3CL1	Fractalkine	P78423	CX3CL1	4.0	4.7	0.85	0
CXCL1	Growth-regulated alpha protein	P09341	CXCL1	0.6	1.2	1.88	0

CXCL5	C-X-C motif chemokine 5	P42830	CXCL5	0.8	1.5	0.69	0
CXCL6	C-X-C motif chemokine 6	P80162	CXCL6	1.1	1.8	0.99	0
CXCL9	C-X-C motif chemokine 9	Q07325	CXCL9	1.3	3.2	1.30	0
CXCL10	C-X-C motif chemokine 10	P02778	CXCL10	1.0	3.1	1.94	0
CXCL11	C-X-C motif chemokine 11	O14625	CXCL11	1.2	3.2	1.52	0
DNER	Delta and Notch-like epidermal growth factor-related receptor	Q8NFT8	DNER	0.9	1.5	0.78	0
EIF4EBP1	Eukaryotic translation initiation factor 4E-binding protein 1	Q13541	EIF4EBP1	1.3	4.9	1.85	0
EN-RAGE	Protein S100-A12 (EN-RAGE)	P80511	S100A12	1.9	9.2	2.35	0
Eotaxin	Eotaxin (CCL11)	P51671	CCL11	1.0	1.7	2.08	0
FGF-5	Fibroblast growth factor 5	P12034	FGF5	4.0	3.8	1.81	2.9
FGF-19	Fibroblast growth factor 19	O95750	FGF19	1.2	2.1	2.28	0
FGF-21	Fibroblast growth factor 21	Q9NSA1	FGF21	1.6	7.0	1.73	0.1
FGF-23*	Fibroblast growth factor 23	Q9GZV9	FGF23	16.8	21.1	0.10	26.1
Flt3L	Fms-related tyrosine kinase 3 ligand	P49771	FLT3LG	1.1	2.0	1.97	0
GDNF	Glial cell line-derived neurotrophic factor	P39905	GDNF	10.6	14.7	0.93	1.3
HGF	Hepatocyte growth factor	P14210	HGF	1.0	1.5	1.74	0
IFNγ	Interferon gamma	P01579	IFNG	1.7	4.0	3.15	0.1
IL-1α*	Interleukin-1 alpha	P01583	IL1A	-15.1	-13.7	-0.38	98.0
IL-2*	Interleukin-2	P60568	IL2	14.6	14.1	1.82	99.5
IL-2RB	Interleukin-2 receptor subunit beta	P14784	IL2RB	11.7	12.1	1.50	2.9
IL-4*	Interleukin-4	P05112	IL4	12.8	15.9	1.57	49.1
IL-5*	Interleukin-5	P05113	IL5	10.7	13.4	1.76	77.6
IL-6	Interleukin-6	P05231	IL6	2.3	5.9	2.13	0
IL-7	Interleukin-7	P13232	IL7	2.2	3.6	0.86	0
IL-8	Interleukin-8	P10145	CXCL8	1.5	2.6	0.77	0
IL-10	Interleukin-10	P22301	IL10	3.7	5.1	1.68	0
IL-10RA	Interleukin-10 receptor subunit alpha	Q13651	IL10RA	4.3	4.9	1.43	12.6

IL-10RB	Interleukin-10 receptor subunit beta	Q08334	IL10RB	1.5	1.8	2.29	0
IL-12B	Interleukin-12 subunit beta	P29460	IL12B	1.4	2.4	0.61	0
IL-13*	Interleukin-13	P35225	IL13	25.5	29.8	0.94	86.2
IL-15RA	Interleukin-15 receptor subunit alpha	Q13261	IL15RA	4.9	6.6	1.40	0.1
IL-17A	Interleukin-17A	Q16552	IL17A	7.1	7.7	1.80	9.4
IL-17C	Interleukin-17C	Q9P0M4	IL17C	4.3	7.6	1.20	0
IL-18	Interleukin-18	Q14116	IL18	1.0	2.0	1.44	0
IL-18R1	Interleukin-18 receptor 1	Q13478	IL18R1	1.1	2.3	1.56	0
IL-20*	Interleukin-20	Q9NYY1	IL20	8.9	8.0	1.64	87.3
IL-20RA*	Interleukin-20 receptor subunit alpha	Q9UHF4	IL20RA	10.3	9.8	1.51	45.2
IL-22 RA1*	Interleukin-22 receptor subunit alpha-1	Q8N6P7	IL22RA1	38.7	29.2	2.43	93.3
IL-24*	Interleukin-24	Q13007	IL24	13.4	12.9	2.86	91.7
IL-33*	Interleukin-33	O95760	IL33	4.0	3.8	2.28	97.3
LAP TGFβ1	Latency-associated peptide transforming growth factor beta-1	P01137	TGFB1	1.7	2.3	1.76	0
LIF*	Leukemia inhibitory factor	P15018	LIF	97.3	40.7	0.59	82.4
LIF-R	Leukemia inhibitory factor receptor	P42702	LIFR	2.3	4.3	1.56	0
MCP-1	Monocyte chemotactic protein 1 (CCL2)	P13500	CCL2	0.8	1.3	1.69	0
MCP-2	Monocyte chemotactic protein 2 (MCP-2, CCL8)	P80075	CCL8	1.0	1.8	1.62	0
MCP-3	Monocyte chemotactic protein 3 (CCL7)	P80098	CCL7	8.3	12.4	0.89	0.1
MCP-4	Monocyte chemotactic protein 4 (CCL13)	Q99616	CCL13	0.8	1.6	2.68	0
MMP-1	Matrix metalloproteinase-1	P03956	MMP1	0.4	1.0	1.70	0
MMP-10	Matrix metalloproteinase-10 (SL-2)	P09238	MMP10	1.0	2.1	1.14	0
Neurturin*	Neurturin	Q99748	NRTN	14.5	13.0	1.37	75.8
NT-3	Neurotrophin-3	P20783	NTF3	5.8	8.2	2.29	5.4
OPG	Osteoprotegerin	O00300	TNFRSF11B	0.8	1.6	1.61	0
OSM	Oncostatin-M	P13725	OSM	1.4	3.4	1.25	0
PD-L1	Programmed cell death 1 ligand 1	Q9NZQ7	CD274	1.9	2.8	1.81	0
SCF	Stem cell factor (c-Kit-ligand)	P21583	KITLG	0.8	2.0	1.41	0

SIRT2	SIR2-like protein 2	Q8IXJ6	SIRT2	3.7	7.5	2.43	2.6
SLAMF1	Signaling lymphocytic activation molecule (SLAM)	Q13291	SLAMF1	5.4	6.6	1.78	0.2
ST1A1	Sulfotransferase 1A1	P50225	SULT1A1	3.0	9.0	1.56	2.1
STAMBP	STAM-binding protein	O95630	STAMBP	2.7	4.2	1.38	0
TGFα	Transforming growth factor alpha	P01135	TGFA	2.7	5.5	0.22	0
TNFα	Tumor necrosis factor-alpha	P01375	TNF	2.3	3.4	0.61	0
TNFβ	Tumor necrosis factor-beta (lymphotoxin-alpha/LT-alpha)	P01374	LTA	2.3	2.4	1.42	0
TNFRSF9	Tumor necrosis factor receptor superfamily member 9	Q07011	TNFRSF9	1.6	2.1	1.15	0
TNFSF14	Tumor necrosis factor ligand superfamily member 14 (LIGHT)	O43557	TNFSF14	1.2	2.9	2.39	0
TRAIL	TNF-related apoptosis-inducing ligand (TNFSF10)	P50591	TNFSF10	1.1	1.9	0.37	0
TRANCE	TNF-related activation-induced cytokine (TRANCE, TNFSF11, RANKL, OPGL)	O14788	TNFSF11	2.3	3.3	1.11	0
TSLP*	Thymic stromal lymphopoietin	Q969D9	TSLP	24.1	26.3	1.86	94.5
TWEAK	Tumor necrosis factor (Ligand) superfamily, member 12 (TWEAK)	O43508	TNFSF12	1.1	1.9	0.70	0
uPA	Urokinase-type plasminogen activator	P00749	PLAU	0.9	1.5	1.62	0
VEGF-A	Vascular endothelial growth factor A	P15692	VEGFA	0.8	1.5	2.02	0

*Excluded from analysis because intra-assay CV >15%, interassay-CV >20% or >25% values below LOD.

LOD, limit of detection; n/a, not applicable (NPX of control measurements below LOD); NPX, normalised protein expression values.

Supplementary Table 3. **Participant characteristics by cohort.**

Characteristics	Study (n=1160)				
	**DIAMOS**	**ECCE HOMO**	**DDCT**	**DIA-LINK1**	**DIA-LINK2**
n	320	244	203	204	189
Age (years)	43.6 ± 14.4	44.8 ± 14.2	45.5 ± 13.6	38.6 ± 12.8	53.2 ± 9.6
Sex , females (%)	165 (51.6)	132 (54.1)	116 (57.1)	120 (58.8)	78 (41.3)
Diabetes type					
Type 1 diabetes	211 (65.9)	159 (65.2)	132 (65.0)	204 (100.0)	0
Type 2 diabetes	109 (34.1)	85 (34.8)	71 (35.0)	0	189 (100.0)
Body mass index (kg/m²)	28.8 ± 6.8	29.8 ± 7.1	29.4 ± 6.8	26.1 ± 5.2	35.6 ± 6.8
HbA1c (%)	8.7 ± 1.8	8.7 ± 1.6	9.3 ± 1.4	8.9 ± 1.9	9.1 ± 1.8
HbA1c (mmol/mol)	72.0 ± 19.2	72.0 ± 17.0	78.1 ± 15.2	71.3 ± 20.4	75.8 ± 19.2
Time since diagnosis of diabetes (years)	14.1 ± 10.2	14.7 ± 10.3	15.6 ± 9.6	18.4 ± 11.8	12.1 ± 7.7
Total cholesterol (mg/dl)	204.5 ± 57.9	190.7 ± 36.0	201.4 ± 46.3	187.3 ± 43.9	176.7 ± 53.9
Triglycerides (mg/dl)	166.6 ± 178.6	156.1 ± 145.5	164.6 ± 118.5	109.5 ± 89.6	219.8 ± 161.1
Lipid-lowering drugs (%)	77 (24.1)	55 (22.5)	47 (23.2)	29 (14.2)	93 (49.2)
NSAIDs (%)	5 (1.6)	9 (3.7)	5 (2.5)	2 (1.0)	1 (0.5)
Antithrombotic drugs (%)	64 (20.0)	39 (16.0)	37 (18.2)	15 (7.4)	40 (21.2)
Antidepressant drugs (%)	13 (4.1)	1 (0.4)	2 (1.0)	19 (9.3)	36 (19.0)
Number of diabetes-related comorbidities	0.8 ± 1.2	0.7 ± 1.0	1.1 ± 1.2	0.7 ± 0.9	1.3 ± 1.4
Retinopathy (%)	69 (21.6)	46 (18.9)	32 (15.8)	49 (24.0)	42 (22.2)
Nephropathy (%)	30 (9.4)	18 (7.4)	20 (9.9)	11 (5.4)	27 (14.3)
Polyneuropathy (%)	101 (31.6)	61 (25.0)	98 (48.3)	66 (32.4)	100 (52.9)
Diabetic foot (%)	8 (2.5)	10 (4.1)	25 (12.3)	3 (1.5)	9 (4.8)
PAOD (%)	16 (5.0)	8 (3.3)	6 (3.0)	6 (2.9)	15 (7.9)
Coronary heart disease (%)	21 (6.6)	20 (8.2)	24 (11.8)	4 (2.0)	22 (11.6)
Myocardial infarction (%)	10 (3.1)	5 (2.0)	11 (5.4)	1 (0.5)	15 (7.9)
Stroke (%)	8 (2.5)	6 (2.5)	3 (1.5)	1 (0.5)	9 (4.8)
CES-D	19.0 ± 11.1	23.7 ± 9.5	24.3 ± 10.8	21.4 ± 11.5	22.2 ± 12.1

Mean ± standard deviation (SD) and n (%) were reported for continuous and categorical variables, respectively.

Diabetes-related comorbidities include retinopathy, diabetes-related chronic kidney disease, polyneuropathy, diabetic foot, PAOD, coronary heart disease, myocardial infarction and stroke (max. 8).

CES-D, Center for Epidemiological Studies-Depression; DDCT, Depression and Diabetes Control Trial; DIA-LINK 1/2, Towards a Better Understanding of Diabetes Distress, Depression and Poor Glycaemic Control in T1D/T2D; DIAMOS, Diabetes Motivation Strengthening; ECCE HOMO, Evaluation of a Stepped Care Approach to Manage Depression in Diabetes; NSAIDs, non-steroidal anti-inflammatory drugs; PAOD, peripheral arterial occlusive disease; T1D, type 1 diabetes; T2D, type 2 diabetes.

Supplementary Table 4. Serum concentrations (in NPX) of biomarkers of inflammation in the study sample (total and stratified by diabetes type).

Biomarkers	Total	T1D	T2D	*P*
ADA	5.45 ± 0.44	5.45 ± 0.45	5.44 ± 0.43	0.238
Axin-1	2.52 ± 0.59	2.52 ± 0.57	2.51 ± 0.61	0.194
Caspase-8	3.96 ± 0.52	3.93 ± 0.50	4.01 ± 0.54	0.007
CCL4	8.17 ± 0.59	8.12 ± 0.59	8.24 ± 0.58	<0.001
CCL19	9.54 ± 0.71	9.45 ± 0.67	9.66 ± 0.76	<0.001
CCL20	7.73 ± 1.12	7.58 ± 0.99	7.94 ± 1.27	<0.001
CCL23	11.07 ± 0.45	11.07 ± 0.44	11.07 ± 0.47	0.864
CCL25	7.20 ± 0.66	7.21 ± 0.67	7.19 ± 0.65	0.900
CCL28	2.63 ± 0.47	2.67 ± 0.49	2.57 ± 0.42	<0.001
CD5	6.62 ± 0.40	6.60 ± 0.38	6.66 ± 0.44	0.023
CD6	5.86 ± 0.51	5.86 ± 0.51	5.85 ± 0.51	0.247
CD8A	10.40 ± 0.68	10.36 ± 0.68	10.46 ± 0.67	0.016
CD244	6.55 ± 0.37	6.57 ± 0.39	6.52 ± 0.33	0.148
CD40	11.76 ± 0.39	11.74 ± 0.37	11.80 ± 0.41	0.003
CDCP1	3.00 ± 0.70	2.82 ± 0.63	3.29 ± 0.72	<0.001
CSF-1	10.38 ± 0.24	10.35 ± 0.23	10.43 ± 0.23	<0.001
CST5	5.73 ± 0.55	5.69 ± 0.55	5.80 ± 0.56	0.001
CX3CL1	4.36 ± 0.43	4.40 ± 0.43	4.31 ± 0.43	<0.001
CXCL1	10.14 ± 0.53	10.13 ± 0.51	10.14 ± 0.57	0.346
CXCL5	12.37 ± 0.64	12.39 ± 0.59	12.33 ± 0.71	0.295
CXCL6	10.33 ± 0.60	10.30 ± 0.59	10.39 ± 0.60	0.014
CXCL9	7.18 ± 0.83	7.12 ± 0.82	7.28 ± 0.83	<0.001
CXCL10	9.93 ± 0.85	9.88 ± 0.80	10.00 ± 0.91	0.008
CXCL11	8.53 ± 0.84	8.52 ± 0.86	8.56 ± 0.81	0.151
DNER	9.09 ± 0.28	9.14 ± 0.26	9.01 ± 0.29	<0.001
EIF4EBP1	7.48 ± 0.99	7.37 ± 1.00	7.65 ± 0.96	<0.001
EN-RAGE	5.58 ± 0.88	5.57 ± 0.87	5.60 ± 0.90	0.689
Eotaxin	9.03 ± 0.49	8.98 ± 0.51	9.11 ± 0.43	<0.001
FGF-5	2.31 ± 0.40	2.31 ± 0.41	2.32 ± 0.37	0.403
FGF-19	8.71 ± 0.94	8.91 ± 0.87	8.41 ± 0.96	<0.001
FGF-21	5.76 ± 1.62	5.22 ± 1.48	6.61 ± 1.46	<0.001
Flt3L	9.37 ± 0.44	9.33 ± 0.44	9.44 ± 0.43	<0.001
GDNF	1.87 ± 0.45	1.85 ± 0.47	1.89 ± 0.42	0.147
HGF	10.30 ± 0.51	10.21 ± 0.49	10.45 ± 0.51	<0.001
IFNγ	6.93 ± 1.14	6.87 ± 1.07	7.01 ± 1.22	0.028
IL-2RB	2.09 ± 0.43	2.10 ± 0.44	2.07 ± 0.42	0.566
IL-6	3.91 ± 0.94	3.69 ± 0.89	4.23 ± 0.91	<0.001
IL-7	4.19 ± 0.52	4.21 ± 0.49	4.16 ± 0.56	0.175

IL-8	6.38 ± 0.70	6.25 ± 0.61	6.58 ± 0.79	<0.001
IL-10	4.00 ± 0.68	3.96 ± 0.61	4.05 ± 0.77	0.239
IL-10RA	1.89 ± 0.63	1.90 ± 0.65	1.86 ± 0.59	0.305
IL-10RB	7.66 ± 0.32	7.63 ± 0.32	7.71 ± 0.33	<0.001
IL-12B	7.58 ± 0.66	7.55 ± 0.63	7.62 ± 0.70	0.067
IL-15RA	2.22 ± 0.30	2.20 ± 0.28	2.27 ± 0.32	<0.001
IL-17A	2.54 ± 0.74	2.50 ± 0.67	2.58 ± 0.83	0.219
IL-17C	3.03 ± 0.84	2.97 ± 0.72	3.11 ± 1.00	0.131
IL-18	9.65 ± 0.56	9.58 ± 0.55	9.75 ± 0.56	<0.001
IL-18R1	8.66 ± 0.48	8.56 ± 0.43	8.80 ± 0.50	<0.001
LAP TGFβ1	9.20 ± 0.39	9.21 ± 0.40	9.18 ± 0.37	0.561
LIF-R	4.07 ± 0.29	4.06 ± 0.29	4.08 ± 0.29	0.627
MCP-1	13.34 ± 0.49	13.29 ± 0.48	13.41 ± 0.49	<0.001
MCP-2	10.66 ± 0.66	10.66 ± 0.65	10.67 ± 0.68	0.775
MCP-3	2.38 ± 0.62	2.23 ± 0.55	2.61 ± 0.66	<0.001
MCP-4	15.18 ± 0.65	15.12 ± 0.66	15.27 ± 0.64	<0.001
MIP-1α	7.60 ± 0.66	7.50 ± 0.64	7.76 ± 0.67	<0.001
MMP-1	15.45 ± 0.73	15.42 ± 0.71	15.49 ± 0.76	0.014
MMP-10	9.47 ± 0.65	9.46 ± 0.67	9.48 ± 0.60	0.150
NT-3	2.84 ± 0.43	2.90 ± 0.44	2.74 ± 0.41	<0.001
OPG	10.45 ± 0.41	10.41 ± 0.39	10.51 ± 0.44	<0.001
OSM	6.80 ± 0.81	6.75 ± 0.80	6.86 ± 0.82	0.078
PD-L1	5.76 ± 0.39	5.75 ± 0.37	5.78 ± 0.41	0.2083
SCF	9.89 ± 0.47	9.94 ± 0.41	9.81 ± 0.54	<0.001
SIRT2	3.49 ± 0.67	3.5 ± 0.69	3.47 ± 0.65	0.593
SLAMF1	2.92 ± 0.44	2.85 ± 0.39	3.03 ± 0.48	<0.001
ST1A1	3.40 ± 1.01	3.46 ± 1.05	3.29 ± 0.94	0.005
STAMBP	4.06 ± 0.51	4.05 ± 0.51	4.08 ± 0.51	0.738
TGFα	4.78 ± 0.71	4.78 ± 0.69	4.78 ± 0.73	0.725
TNFRSF9	6.59 ± 0.52	6.58 ± 0.46	6.61 ± 0.59	0.725
TNFSF14	7.24 ± 0.67	7.25 ± 0.67	7.21 ± 0.67	0.191
TNFα	4.17 ± 0.51	4.11 ± 0.50	4.26 ± 0.50	<0.001
TNFβ	5.25 ± 0.48	5.28 ± 0.43	5.20 ± 0.53	<0.001
TRAIL	7.97 ± 0.32	7.98 ± 0.33	7.95 ± 0.30	0.060
TRANCE	5.22 ± 0.68	5.29 ± 0.68	5.11 ± 0.67	<0.001
TWEAK	9.51 ± 0.39	9.61 ± 0.36	9.37 ± 0.38	<0.001
uPA	10.40 ± 0.35	10.39 ± 0.34	10.4 ± 0.36	0.764
VEGF-A	12.17 ± 0.56	12.13 ± 0.56	12.24 ± 0.54	<0.001

NPX, normalised protein expression values.

Supplementary Table 5. Associations of biomarkers of inflammation and the total CES-D score (model 1)

Biomarkers	Total		T1D		T2D		$P_{interaction}$
	β	*P*	β	*P*	β	*P*	
ADA	0.032	0.263	0.010	0.779	0.085	0.079	0.229
Axin-1	0.024	0.394	-0.002	0.949	0.049	0.265	0.413
Caspase-8	**0.056**	**0.049**	0.048	0.195	0.058	0.198	0.820
CCL4	0.010	0.715	-0.041	0.244	0.082	0.082	0.050
CCL19	-0.016	0.579	-0.057	0.125	0.047	0.288	0.097
CCL20	0.026	0.358	0.020	0.603	0.030	0.454	0.824
CCL23	0.007	0.807	0.021	0.544	0.007	0.871	0.577
CCL25	**0.093**	**0.001**	**0.082**	**0.018**	**0.120**	**0.011**	0.833
CCL28	0.007	0.800	-0.006	0.849	0.044	0.396	0.467
CD5	0.051	0.068	0.025	0.502	**0.098**	**0.022**	0.156
CD6	0.015	0.583	-0.031	0.372	**0.097**	**0.035**	**0.013**
CD8A	0.054	0.058	0.043	0.236	0.082	0.079	0.243
CD40	**0.059**	**0.039**	0.039	0.297	**0.092**	**0.036**	0.667
CD244	0.012	0.652	-0.028	0.400	**0.115**	**0.024**	**0.014**
CDCP1	**0.098**	**0.002**	0.081	0.056	**0.112**	**0.019**	0.882
CSF-1	0.040	0.160	0.021	0.562	0.077	0.109	0.282
CST5	0.025	0.390	0.026	0.477	0.032	0.501	0.507
CX3CL1	0.019	0.506	0.006	0.861	0.054	0.249	0.619
CXCL1	**0.058**	**0.041**	0.045	0.217	0.067	0.137	0.439
CXCL5	0.039	0.192	0.054	0.176	0.012	0.787	0.997
CXCL6	0.045	0.111	0.015	0.673	**0.099**	**0.035**	0.078
CXCL9	0.012	0.693	0.029	0.421	0.010	0.830	0.222
CXCL10	-0.027	0.348	0.012	0.739	-0.069	0.112	0.081
CXCL11	-0.010	0.730	0.003	0.929	-0.024	0.614	0.717
DNER	0.005	0.862	-0.023	0.550	0.022	0.634	0.382
EIF4EBP1	0.049	0.086	0.050	0.158	0.055	0.246	0.788
EN-RAGE	**0.057**	**0.040**	0.036	0.312	0.081	0.074	0.381
Eotaxin	**0.076**	**0.011**	**0.075**	**0.042**	0.052	0.321	0.403
FGF-5	0.016	0.567	0.059	0.087	-0.058	0.243	**0.015**
FGF-19	0.053	0.068	0.034	0.357	0.084	0.069	0.341
FGF-21	**0.116**	**<0.001**	**0.094**	**0.013**	**0.161**	**0.004**	0.360
Flt3L	0.044	0.135	0.066	0.068	0.002	0.963	0.204
GDNF	0.047	0.091	-0.006	0.853	**0.141**	**0.004**	**0.020**
HGF	**0.068**	**0.018**	0.060	0.097	0.066	0.162	0.756
IFNγ	-0.001	0.963	0.057	0.122	-0.064	0.139	0.018
IL-2RB	0.028	0.303	0.015	0.656	0.060	0.206	0.521
IL-6	0.032	0.291	0.030	0.436	0.027	0.582	0.785

IL-7	-0.015	0.589	-0.005	0.902	-0.042	0.325	0.699
IL-8	**0.096**	**0.001**	**0.089**	**0.028**	**0.100**	**0.015**	0.936
IL-10	0.024	0.390	0.032	0.407	0.022	0.594	0.745
IL-10RA	0.006	0.839	0.005	0.877	0.004	0.940	0.876
IL-10RB	**0.085**	**0.002**	0.063	0.075	**0.136**	**0.003**	0.269
IL-12B	0.010	0.739	-0.033	0.382	0.085	0.057	0.070
IL-15RA	0.052	0.065	0.025	0.495	**0.102**	**0.020**	0.504
IL-17A	0.037	0.180	0.004	0.912	0.070	0.084	0.200
IL-17C	-0.001	0.984	-0.005	0.906	0.005	0.891	0.966
IL-18	**0.076**	**0.007**	**0.072**	**0.044**	**0.092**	**0.046**	0.868
IL-18R1	**0.070**	**0.015**	-0.009	0.816	**0.157**	**<0.001**	**0.003**
LAP TGFβ1	-0.004	0.889	-0.021	0.535	0.017	0.734	0.281
LIF-R	0.038	0.172	-0.029	0.406	**0.156**	**0.001**	**0.003**
MCP-1	0.047	0.095	0.047	0.187	0.034	0.474	0.606
MCP-2	-0.013	0.626	-0.039	0.261	0.022	0.624	0.254
MCP-3	**0.068**	**0.025**	0.077	0.060	0.040	0.386	0.483
MCP-4	0.028	0.333	0.007	0.854	0.037	0.444	0.728
MIP-1α	0.054	0.058	0.022	0.544	**0.099**	**0.032**	0.301
MMP-1	0.037	0.182	0.031	0.392	0.029	0.523	0.946
MMP-10	**0.081**	**0.004**	**0.091**	**0.006**	0.086	0.088	0.580
NT-3	-0.019	0.502	-0.056	0.110	0.046	0.351	**0.024**
OPG	0.022	0.472	-0.045	0.257	**0.112**	**0.016**	**0.049**
OSM	0.042	0.130	0.062	0.080	0.002	0.961	0.611
PD-L1	0.017	0.546	0.014	0.697	0.034	0.439	0.947
SCF	-0.033	0.246	-0.001	0.972	-0.061	0.127	0.207
SIRT2	0.033	0.237	0.009	0.794	0.079	0.095	0.213
SLAMF1	0.038	0.184	0.039	0.320	0.042	0.310	0.750
ST1A1	0.006	0.841	0.014	0.683	-0.005	0.919	0.799
STAMBP	0.037	0.179	0.018	0.617	0.074	0.102	0.419
TGFα	0.045	0.103	0.049	0.172	0.034	0.457	0.816
TNFRSF9	**0.090**	**0.001**	**0.105**	**0.007**	**0.103**	**0.012**	0.577
TNFSF14	0.027	0.334	0.030	0.388	0.006	0.904	0.961
TNFα	0.034	0.221	0.035	0.324	0.044	0.349	0.854
TNFβ	-0.013	0.659	**-0.079**	**0.049**	0.053	0.210	**0.006**
TRAIL	0.037	0.183	0.061	0.083	-0.025	0.601	0.178
TRANCE	0.031	0.294	0.055	0.139	-0.006	0.903	0.532
TWEAK	-0.025	0.403	-0.053	0.157	-0.001	0.992	0.232
uPA	0.036	0.204	0.016	0.669	0.051	0.251	0.638
VEGF-A	0.036	0.193	0.033	0.339	0.041	0.390	0.904

Model 1 was adjusted for age, sex and study cohort.

T1D, type 1 diabetes. T2D, type 2 diabetes; Total, people with type 1 or 2 diabetes.

See **Supplementary Table 2** for a list of full biomarker names.

Supplementary Table 6. Associations of biomarkers of inflammation and the total CES-D score (model 2)

Biomarkers	Total		T1D		T2D		$P_{interaction}$
	**β**	***P***	**β**	***P***	**β**	***P***	
ADA	0.013	0.633	-0.005	0.880	0.043	0.376	0.436
Axin-1	0.017	0.547	-0.009	0.798	0.041	0.344	0.493
Caspase-8	0.036	0.203	0.033	0.363	0.035	0.441	0.794
CCL4	-0.007	0.804	-0.044	0.207	0.054	0.257	0.055
CCL19	-0.022	0.439	-0.050	0.164	0.033	0.473	0.130
CCL20	0.010	0.715	0.010	0.792	0.009	0.823	0.980
CCL23	0.009	0.752	0.027	0.430	-0.001	0.984	0.404
CCL25	**0.076**	**0.007**	0.060	0.082	**0.121**	**0.011**	0.705
CCL28	0.010	0.723	-0.011	0.753	0.057	0.276	0.356
CD5	0.038	0.172	0.012	0.734	0.082	0.057	0.176
CD6	0.002	0.935	-0.040	0.231	0.073	0.113	**0.017**
CD8A	0.041	0.145	0.020	0.562	0.072	0.127	0.217
CD40	0.033	0.251	0.011	0.763	0.074	0.097	0.675
CD244	0.007	0.793	-0.028	0.371	0.097	0.059	**0.028**
CDCP1	**0.067**	**0.038**	0.063	0.146	0.068	0.184	0.835
CSF-1	0.015	0.599	-0.008	0.828	0.058	0.248	0.198
CST5	0.021	0.456	0.024	0.495	0.014	0.772	0.457
CX3CL1	0.0001	0.998	-0.031	0.364	0.037	0.429	0.460
CXCL1	0.035	0.210	0.033	0.360	0.043	0.340	0.600
CXCL5	0.031	0.282	0.048	0.217	0.009	0.836	0.961
CXCL6	0.034	0.219	0.008	0.818	0.087	0.060	0.092
CXCL9	0.006	0.836	0.024	0.502	0.014	0.782	0.245
CXCL10	-0.031	0.272	0.003	0.924	-0.067	0.127	0.148
CXCL11	-0.018	0.506	-0.005	0.884	-0.021	0.658	0.695
DNER	0.004	0.888	-0.034	0.382	0.023	0.629	0.348
EIF4EBP1	0.017	0.557	0.021	0.536	0.011	0.816	0.583
EN-RAGE	**0.054**	**0.049**	0.037	0.287	0.070	0.116	0.467
Eotaxin	**0.071**	**0.018**	**0.076**	**0.040**	0.045	0.390	0.419
FGF-5	0.017	0.538	0.056	0.100	-0.060	0.234	**0.019**
FGF-19	0.044	0.130	0.012	0.735	0.083	0.073	0.341
FGF-21	**0.081**	**0.017**	0.075	0.064	**0.141**	**0.028**	0.368
Flt3L	0.013	0.661	0.045	0.219	-0.046	0.351	0.083
GDNF	0.048	0.084	-0.006	0.854	**0.147**	**0.002**	**0.041**
HGF	0.053	0.067	0.054	0.130	0.047	0.338	0.820
IFNγ	-0.008	0.769	0.051	0.161	-0.060	0.163	**0.020**
IL-2RB	0.029	0.291	0.017	0.595	0.057	0.227	0.610
IL-6	0.022	0.473	0.027	0.476	0.012	0.823	0.818

IL-7	-0.023	0.400	-0.012	0.746	-0.036	0.400	0.884
IL-8	**0.064**	**0.025**	0.054	0.179	0.070	0.097	0.927
IL-10	0.017	0.541	0.029	0.449	0.020	0.618	0.878
IL-10RA	0.001	0.960	0.012	0.720	0.007	0.888	0.849
IL-10RB	**0.062**	**0.027**	0.038	0.285	**0.117**	**0.014**	0.241
IL-12B	0.003	0.925	-0.033	0.380	0.082	0.076	0.108
IL-15RA	0.030	0.284	0.001	0.989	0.083	0.062	0.410
IL-17A	0.037	0.172	0.003	0.940	0.067	0.097	0.201
IL-17C	-0.001	0.965	0.009	0.828	-0.001	0.973	0.831
IL-18	**0.064**	**0.024**	0.058	0.098	0.083	0.080	0.565
IL-18R1	0.048	0.099	-0.021	0.586	**0.134**	**0.003**	**0.003**
LAP TGFβ1	-0.014	0.615	-0.033	0.321	0.002	0.962	0.286
LIF-R	0.020	0.470	-0.046	0.182	**0.125**	**0.008**	**0.011**
MCP-1	0.020	0.474	0.026	0.461	0.006	0.897	0.603
MCP-2	-0.031	0.250	-0.048	0.165	0.005	0.913	0.267
MCP-3	0.041	0.202	0.062	0.135	0.0001	0.998	0.424
MCP-4	0.016	0.577	-0.002	0.962	0.035	0.468	0.561
MIP-1α	0.033	0.247	0.012	0.734	0.075	0.110	0.279
MMP-1	0.030	0.272	0.021	0.541	0.029	0.510	0.779
MMP-10	**0.066**	**0.018**	**0.079**	**0.017**	0.069	0.180	0.359
NT-3	-0.031	0.268	-0.058	0.092	0.017	0.738	0.118
OPG	0.008	0.799	-0.062	0.114	0.087	0.062	0.042
OSM	0.036	0.190	0.060	0.086	0.0002	0.996	0.635
PD-L1	0.004	0.898	0.003	0.926	0.017	0.697	0.976
SCF	-0.014	0.646	-0.015	0.727	-0.025	0.551	0.372
SIRT2	0.011	0.698	-0.013	0.694	0.048	0.312	0.309
SLAMF1	0.024	0.392	0.022	0.576	0.028	0.498	0.907
ST1A1	-0.005	0.842	-0.00002	0.999	-0.021	0.671	0.616
STAMBP	0.013	0.629	-0.004	0.906	0.044	0.336	0.534
TGFα	0.042	0.123	0.044	0.201	0.037	0.410	0.753
TNFRSF9	**0.077**	**0.005**	**0.075**	**0.047**	**0.094**	**0.024**	0.666
TNFSF14	0.021	0.433	0.038	0.260	-0.009	0.843	0.787
TNFα	0.019	0.510	0.029	0.399	0.016	0.735	0.806
TNFβ	-0.015	0.593	**-0.088**	**0.024**	0.038	0.366	**0.011**
TRAIL	0.023	0.408	0.058	0.090	-0.030	0.533	0.137
TRANCE	0.024	0.415	0.052	0.161	-0.008	0.875	0.493
TWEAK	-0.016	0.591	-0.053	0.147	0.013	0.794	0.155
uPA	0.017	0.544	-0.003	0.938	0.022	0.624	0.950
VEGF-A	0.010	0.729	0.017	0.621	0.011	0.816	0.922

Model 2 was adjusted for age, sex, study cohort, BMI, HbA1c, diabetes duration, total cholesterol, triglycerides, use of lipid-lowering drugs, use of NSAIDs, use of antithrombotic medication and use of antidepressant medication.

T1D, type 1 diabetes. T2D, type 2 diabetes; Total, people with type 1 or 2 diabetes.

See **Supplementary Table 2** for a list of full biomarker names.

Supplementary Table 7. Associations of biomarkers of inflammation and the total CES-D score (model 3)

Biomarkers	Total		T1D		T2D		$P_{interaction}$
	β	*P*	β	*P*	β	*P*	
ADA	0.010	0.718	-0.003	0.931	0.036	0.456	0.437
Axin-1	0.016	0.564	-0.009	0.798	0.037	0.392	0.507
Caspase-8	0.034	0.230	0.033	0.352	0.026	0.568	0.760
CCL4	-0.008	0.772	-0.043	0.208	0.046	0.328	0.061
CCL19	-0.025	0.381	-0.050	0.168	0.017	0.702	0.156
CCL20	0.009	0.754	0.011	0.779	0.005	0.913	0.967
CCL23	0.005	0.858	0.029	0.402	-0.012	0.781	0.383
CCL25	**0.073**	**0.009**	0.062	0.077	**0.109**	**0.022**	0.761
CCL28	0.010	0.733	-0.011	0.753	0.054	0.297	0.366
CD5	0.033	0.248	0.017	0.655	0.067	0.122	0.180
CD6	0.001	0.967	-0.040	0.242	0.071	0.120	**0.016**
CD8A	0.041	0.151	0.021	0.546	0.072	0.124	0.203
CD40	0.026	0.370	0.016	0.684	0.054	0.239	0.726
CD244	0.006	0.823	-0.028	0.379	0.092	0.069	**0.029**
CDCP1	**0.065**	**0.045**	0.063	0.141	0.055	0.275	0.781
CSF-1	0.010	0.742	-0.005	0.886	0.036	0.474	0.222
CST5	0.018	0.519	0.025	0.476	0.004	0.934	0.423
CX3CL1	-0.006	0.843	-0.029	0.406	0.023	0.622	0.474
CXCL1	0.034	0.230	0.034	0.350	0.036	0.422	0.610
CXCL5	0.032	0.276	0.048	0.225	0.005	0.907	0.976
CXCL6	0.033	0.236	0.008	0.807	0.080	0.082	0.095
CXCL9	0.003	0.915	0.025	0.484	0.001	0.982	0.212
CXCL10	-0.030	0.285	0.003	0.939	-0.068	0.120	0.138
CXCL11	-0.018	0.515	-0.005	0.881	-0.023	0.629	0.687
DNER	0.006	0.855	-0.035	0.375	0.024	0.613	0.341
EIF4EBP1	0.013	0.654	0.023	0.509	-0.006	0.901	0.519
EN-RAGE	0.053	0.053	0.037	0.286	0.064	0.149	0.486
Eotaxin	**0.070**	**0.019**	**0.077**	**0.037**	0.043	0.406	0.420
FGF-5	0.014	0.626	0.058	0.088	-0.073	0.147	**0.016**
FGF-19	0.043	0.134	0.013	0.724	0.084	0.068	0.328
FGF-21	**0.078**	**0.021**	0.076	0.062	0.123	0.055	0.413
Flt3L	0.012	0.671	0.045	0.220	-0.050	0.309	0.075
GDNF	0.045	0.101	-0.005	0.886	**0.141**	**0.004**	**0.043**
HGF	0.051	0.079	0.055	0.123	0.036	0.465	0.853
IFNγ	-0.008	0.762	0.051	0.159	-0.062	0.146	**0.020**
IL-2RB	0.029	0.291	0.017	0.606	0.055	0.244	0.639
IL-6	0.019	0.537	0.029	0.450	-0.003	0.948	0.771

IL-7	-0.021	0.448	-0.013	0.729	-0.030	0.485	0.903
IL-8	**0.063**	**0.028**	0.056	0.164	0.070	0.099	0.947
IL-10	0.016	0.562	0.029	0.444	0.017	0.661	0.874
IL-10RA	0.001	0.979	0.013	0.689	0.013	0.793	0.781
IL-10RB	**0.057**	**0.046**	0.043	0.238	**0.097**	**0.043**	0.265
IL-12B	-0.002	0.945	-0.031	0.407	0.068	0.143	0.120
IL-15RA	0.024	0.393	0.003	0.927	0.064	0.156	0.459
IL-17A	0.036	0.193	0.005	0.895	0.065	0.102	0.188
IL-17C	-0.004	0.894	0.011	0.789	-0.005	0.897	0.852
IL-18	**0.063**	**0.026**	0.059	0.096	0.078	0.099	0.581
IL-18R1	0.045	0.129	-0.019	0.615	**0.121**	**0.008**	**0.003**
LAP TGFβ1	-0.013	0.644	-0.033	0.316	0.007	0.887	0.273
LIF-R	0.018	0.512	-0.045	0.191	**0.121**	**0.010**	**0.012**
MCP-1	0.019	0.499	0.027	0.452	-0.001	0.976	0.580
MCP-2	-0.032	0.240	-0.048	0.168	0.000	0.996	0.269
MCP-3	0.040	0.215	0.063	0.127	-0.003	0.946	0.424
MCP-4	0.015	0.603	-0.002	0.965	0.025	0.598	0.606
MIP-1α	0.031	0.289	0.013	0.717	0.062	0.190	0.311
MMP-1	0.030	0.275	0.022	0.530	0.027	0.545	0.779
MMP-10	**0.063**	**0.023**	**0.080**	**0.015**	0.055	0.280	0.316
NT-3	-0.034	0.238	-0.057	0.095	0.010	0.845	0.122
OPG	0.003	0.909	-0.061	0.121	0.072	0.121	0.050
OSM	0.035	0.208	0.060	0.084	-0.008	0.865	0.622
PD-L1	-0.002	0.949	0.006	0.868	0.001	0.985	0.937
SCF	-0.016	0.586	-0.013	0.756	-0.030	0.470	0.368
SIRT2	0.009	0.732	-0.013	0.704	0.043	0.362	0.318
SLAMF1	0.020	0.481	0.024	0.532	0.016	0.702	0.879
ST1A1	-0.005	0.868	0.000	0.989	-0.017	0.734	0.638
STAMBP	0.011	0.690	-0.003	0.923	0.035	0.439	0.564
TGFα	0.041	0.138	0.045	0.198	0.028	0.530	0.777
TNFRSF9	**0.072**	**0.011**	**0.083**	**0.033**	0.072	0.089	0.631
TNFSF14	0.020	0.455	0.038	0.255	-0.014	0.760	0.792
TNFα	0.016	0.572	0.031	0.378	0.007	0.887	0.774
TNFβ	-0.015	0.592	**-0.088**	**0.024**	0.037	0.366	**0.011**
TRAIL	0.021	0.461	0.060	0.081	-0.042	0.388	0.135
TRANCE	0.023	0.446	0.053	0.155	-0.015	0.760	0.488
TWEAK	-0.014	0.631	-0.053	0.146	0.020	0.673	0.138
uPA	0.015	0.588	-0.003	0.944	0.011	0.815	0.991
VEGF-A	0.007	0.810	0.017	0.608	-0.006	0.908	0.838

Model 3 was adjusted for age, sex, study cohort, BMI, HbA1c, diabetes duration, total cholesterol, triglycerides, use of lipid-lowering drugs, use of NSAIDs, use of

antithrombotic medication, use of antidepressant medication and the number of diabetes-related comorbidities.

T1D, type 1 diabetes; T2D, type 2 diabetes; Total, people with type 1 or 2 diabetes.

See **Supplementary Table 2** for a list of full biomarker names.

Supplementary Table 8. Associations of biomarkers of inflammation and cognitive-affective symptoms in the CES-D score (model 3)

Biomarkers	Total		T1D		T2D		$P_{interaction}$
	β	*P*	β	*P*	β	*P*	
ADA	0.011	0.697	-0.004	0.912	0.042	0.399	0.382
Axin-1	0.018	0.525	0.003	0.929	0.030	0.502	0.725
Caspase-8	0.032	0.267	0.032	0.373	0.025	0.586	0.779
CCL4	-0.021	0.471	-0.062	0.074	0.046	0.346	**0.032**
CCL19	-0.027	0.349	-0.047	0.193	0.006	0.901	0.269
CCL20	0.009	0.742	0.011	0.767	0.005	0.914	0.880
CCL23	0.015	0.591	0.045	0.199	-0.010	0.834	0.320
CCL25	**0.075**	**0.009**	0.059	0.093	**0.115**	**0.020**	0.604
CCL28	0.037	0.208	0.020	0.564	0.070	0.195	0.457
CD5	0.018	0.544	0.001	0.970	0.049	0.283	0.255
CD6	-0.006	0.830	-0.042	0.221	0.054	0.258	**0.046**
CD8A	0.024	0.397	0.012	0.736	0.044	0.368	0.486
CD40	0.012	0.688	0.005	0.901	0.035	0.455	0.743
CD244	-0.002	0.955	-0.021	0.506	0.053	0.318	0.184
CDCP1	0.063	0.057	0.053	0.222	0.064	0.225	0.920
CSF-1	0.004	0.895	-0.003	0.925	0.014	0.783	0.468
CST5	0.040	0.169	0.054	0.130	0.017	0.723	0.347
CX3CL1	-0.006	0.820	-0.020	0.570	0.009	0.855	0.650
CXCL1	0.037	0.196	0.046	0.208	0.030	0.517	0.991
CXCL5	**0.061**	**0.038**	0.076	0.056	0.040	0.375	0.932
CXCL6	0.037	0.192	0.027	0.446	0.060	0.210	0.402
CXCL9	0.009	0.761	0.030	0.403	0.004	0.935	0.241
CXCL10	-0.022	0.443	-0.0004	0.991	-0.047	0.304	0.294
CXCL11	-0.008	0.765	0.002	0.960	-0.014	0.780	0.643
DNER	0.013	0.671	-0.026	0.516	0.042	0.396	0.252
EIF4EBP1	-0.011	0.707	0.003	0.928	-0.041	0.428	0.374
EN-RAGE	0.053	0.057	0.049	0.157	0.052	0.258	0.826
Eotaxin	**0.069**	**0.023**	0.066	0.078	0.074	0.176	0.917
FGF-5	0.026	0.364	0.063	0.069	-0.046	0.380	0.069
FGF-19	0.050	0.088	0.040	0.283	0.066	0.169	0.803
FGF-21	**0.069**	**0.045**	0.077	0.061	0.076	0.253	0.977
Flt3L	-0.005	0.869	0.028	0.444	-0.067	0.192	0.069
GDNF	0.029	0.299	-0.012	0.714	**0.111**	**0.028**	0.086
HGF	0.041	0.169	0.049	0.175	0.024	0.641	0.932
IFNγ	-0.013	0.638	0.026	0.480	-0.046	0.307	0.115
IL-2RB	0.024	0.385	0.020	0.557	0.031	0.520	0.901
IL-6	-0.006	0.862	0.008	0.844	-0.037	0.503	0.673

IL-7	-0.001	0.985	0.004	0.910	-0.007	0.873	0.935
IL-8	0.055	0.059	0.049	0.231	0.064	0.142	0.910
IL-10	-0.014	0.622	-0.001	0.969	-0.015	0.723	0.856
IL-10RA	0.005	0.864	0.018	0.580	0.018	0.717	0.838
IL-10RB	0.056	0.054	0.059	0.106	0.065	0.191	0.746
IL-12B	-0.006	0.839	-0.023	0.550	0.045	0.348	0.314
IL-15RA	0.023	0.429	-0.003	0.937	0.067	0.151	0.335
IL-17A	0.034	0.225	0.012	0.746	0.054	0.197	0.378
IL-17C	0.006	0.836	0.025	0.531	-0.0004	0.991	0.758
IL-18	0.055	0.058	0.039	0.272	0.085	0.087	0.393
IL-18R1	0.022	0.470	-0.027	0.487	0.080	0.091	**0.026**
LAP TGFβ1	-0.007	0.815	-0.026	0.438	0.013	0.796	0.360
LIF-R	-0.002	0.954	-0.042	0.220	0.067	0.172	0.124
MCP-1	0.020	0.475	0.047	0.185	-0.021	0.664	0.215
MCP-2	-0.024	0.385	-0.047	0.180	0.015	0.752	0.200
MCP-3	0.027	0.414	0.054	0.196	-0.018	0.723	0.344
MCP-4	0.035	0.221	0.030	0.401	0.036	0.471	0.851
MIP-1α	0.010	0.726	-0.008	0.832	0.042	0.395	0.332
MMP-1	0.041	0.136	0.051	0.150	0.014	0.764	0.583
MMP-10	0.045	0.110	0.064	0.056	0.031	0.566	0.273
NT-3	-0.024	0.401	-0.036	0.295	0.006	0.906	0.317
OPG	-0.004	0.898	-0.055	0.166	0.051	0.292	0.108
OSM	0.014	0.620	0.043	0.222	-0.033	0.489	0.413
PD-L1	-0.011	0.703	-0.002	0.949	-0.009	0.848	0.972
SCF	0.018	0.547	0.038	0.377	-0.002	0.965	0.341
SIRT2	-0.0002	0.993	-0.012	0.726	0.014	0.778	0.681
SLAMF1	-0.001	0.979	-0.003	0.936	0.0001	0.999	0.920
ST1A1	-0.002	0.950	-0.0003	0.993	-0.009	0.856	0.725
STAMBP	0.007	0.799	-0.003	0.940	0.023	0.621	0.740
TGFα	0.021	0.452	0.027	0.448	0.006	0.903	0.941
TNFRSF9	**0.057**	**0.049**	0.063	0.105	0.056	0.208	0.729
TNFSF14	0.003	0.928	0.026	0.450	-0.034	0.477	0.583
TNFα	-0.001	0.961	0.021	0.558	-0.023	0.641	0.519
TNFβ	-0.025	0.383	-0.058	0.138	-0.007	0.863	0.248
TRAIL	0.021	0.450	0.044	0.211	-0.010	0.850	0.544
TRANCE	0.038	0.217	0.060	0.107	0.007	0.888	0.612
TWEAK	0.0004	0.989	-0.034	0.351	0.036	0.469	0.146
uPA	-0.004	0.891	-0.002	0.958	-0.030	0.521	0.544
VEGF-A	0.017	0.546	0.033	0.328	-0.010	0.838	0.538

Model 3 was adjusted for age, sex, study cohort, BMI, HbA1c, diabetes duration, total cholesterol, triglycerides, use of lipid-lowering drugs, use of NSAIDs, use of

antithrombotic medication, use of antidepressant medication and the number of diabetes-related comorbidities.

T1D, type 1 diabetes; T2D, type 2 diabetes; Total, people with type 1 or 2 diabetes.

See **Supplementary Table 2** for a list of full biomarker names.

Supplementary Table 9. Associations of biomarkers of inflammation and somatic symptoms in the CES-D score (model 3)

Biomarkers	Total		T1D		T2D		$P_{interaction}$
	β	*P*	β	*P*	β	*P*	
ADA	0.007	0.813	-0.002	0.947	0.027	0.578	0.573
Axin-1	-0.008	0.763	-0.016	0.657	-0.013	0.754	0.858
Caspase-8	0.017	0.561	0.037	0.319	-0.022	0.623	0.224
CCL4	0.014	0.633	-0.001	0.985	0.033	0.477	0.407
CCL19	-0.024	0.403	-0.043	0.252	0.014	0.758	0.219
CCL20	0.027	0.349	0.026	0.509	0.024	0.555	0.908
CCL23	-0.004	0.879	0.038	0.291	-0.049	0.270	0.096
CCL25	**0.075**	**0.009**	0.070	0.053	**0.100**	**0.034**	0.984
CCL28	-0.023	0.434	-0.046	0.191	0.028	0.584	0.286
CD5	0.031	0.279	0.022	0.580	0.058	0.181	0.259
CD6	0.005	0.867	-0.031	0.374	0.070	0.124	**0.028**
CD8A	0.034	0.233	0.026	0.470	0.048	0.302	0.372
CD40	0.037	0.211	0.042	0.286	0.043	0.342	0.751
CD244	0.021	0.454	-0.005	0.891	0.091	0.069	0.080
CDCP1	0.065	0.051	**0.094**	**0.036**	0.023	0.650	0.252
CSF-1	0.019	0.532	0.008	0.822	0.039	0.440	0.307
CST5	0.002	0.952	0.007	0.848	-0.011	0.815	0.424
CX3CL1	0.005	0.868	-0.006	0.860	0.014	0.768	0.939
CXCL1	0.014	0.632	0.015	0.689	0.014	0.763	0.615
CXCL5	0.005	0.875	0.045	0.277	-0.049	0.250	0.493
CXCL6	0.017	0.556	-0.004	0.923	0.062	0.178	0.122
CXCL9	0.007	0.826	0.015	0.686	0.032	0.510	0.546
CXCL10	-0.030	0.285	0.014	0.712	-0.079	0.068	0.069
CXCL11	-0.037	0.186	-0.029	0.412	-0.025	0.597	0.975
DNER	-0.018	0.562	-0.058	0.159	-0.006	0.898	0.457
EIF4EBP1	0.016	0.592	0.035	0.337	-0.016	0.750	0.308
EN-RAGE	0.044	0.117	0.029	0.417	0.049	0.267	0.559
Eotaxin	**0.062**	**0.042**	0.064	0.097	0.037	0.481	0.483
FGF-5	0.038	0.194	**0.090**	**0.012**	-0.061	0.221	**0.006**
FGF-19	0.056	0.058	0.015	0.702	**0.108**	**0.017**	0.180
FGF-21	**0.084**	**0.015**	0.076	0.072	**0.142**	**0.026**	0.263
Flt3L	0.038	0.202	0.068	0.073	-0.020	0.681	0.112
GDNF	0.052	0.065	0.014	0.689	**0.125**	**0.009**	0.145
HGF	0.038	0.194	0.039	0.294	0.026	0.595	0.734
IFNγ	-0.002	0.932	0.063	0.096	-0.057	0.182	**0.016**
IL-2RB	0.034	0.225	0.022	0.515	0.060	0.198	0.675
IL-6	0.036	0.253	0.043	0.281	0.022	0.676	0.809

IL-7	-0.048	0.086	-0.026	0.490	-0.073	0.083	0.540
IL-8	0.052	0.077	0.064	0.128	0.038	0.365	0.511
IL-10	0.019	0.498	0.019	0.627	0.034	0.387	0.802
IL-10RA	0.011	0.678	0.020	0.567	0.024	0.617	0.707
IL-10RB	0.053	0.069	0.049	0.191	0.077	0.105	0.502
IL-12B	-0.0001	0.999	-0.021	0.592	0.061	0.181	0.192
IL-15RA	0.032	0.277	0.036	0.353	0.039	0.383	0.791
IL-17A	0.024	0.387	-0.016	0.682	0.064	0.104	0.081
IL-17C	0.016	0.565	0.038	0.365	0.008	0.834	0.656
IL-18	**0.070**	**0.016**	0.072	0.051	0.078	0.096	0.703
IL-18R1	0.044	0.139	0.010	0.811	0.084	0.064	0.060
LAP TGFβ1	-0.016	0.566	-0.024	0.485	-0.021	0.667	0.609
LIF-R	0.026	0.353	-0.029	0.420	**0.114**	**0.014**	**0.037**
MCP-1	0.020	0.493	0.016	0.658	0.012	0.803	0.817
MCP-2	-0.017	0.553	-0.013	0.726	-0.008	0.862	0.780
MCP-3	0.053	0.101	0.074	0.087	0.016	0.741	0.486
MCP-4	0.001	0.961	-0.004	0.910	-0.009	0.842	0.954
MIP-1α	0.031	0.289	0.032	0.394	0.036	0.440	0.837
MMP-1	0.013	0.636	-0.019	0.606	0.046	0.290	0.168
MMP-10	**0.069**	**0.016**	**0.084**	**0.015**	0.064	0.204	0.388
NT-3	-0.015	0.598	-0.035	0.325	0.013	0.786	0.201
OPG	0.017	0.570	-0.039	0.342	0.076	0.100	0.117
OSM	0.033	0.244	0.051	0.164	0.001	0.978	0.918
PD-L1	0.014	0.614	0.018	0.638	0.025	0.570	0.973
SCF	-0.025	0.405	-0.030	0.508	-0.039	0.350	0.392
SIRT2	0.001	0.967	-0.004	0.914	0.006	0.906	0.887
SLAMF1	0.038	0.187	0.051	0.208	0.029	0.479	0.659
ST1A1	-0.018	0.510	-0.002	0.946	-0.056	0.248	0.255
STAMBP	-0.003	0.921	0.002	0.948	-0.009	0.838	0.732
TGFα	0.035	0.209	0.027	0.457	0.039	0.375	0.418
TNFRSF9	**0.081**	**0.005**	**0.099**	**0.014**	0.076	0.071	0.494
TNFSF14	0.006	0.823	0.026	0.467	-0.035	0.438	0.707
TNFα	0.036	0.208	0.043	0.233	0.042	0.366	0.957
TNFβ	-0.004	0.898	**-0.093**	**0.022**	0.064	0.119	**0.002**
TRAIL	0.036	0.205	**0.092**	**0.011**	-0.049	0.308	**0.026**
TRANCE	0.025	0.418	0.058	0.133	-0.014	0.776	0.411
TWEAK	-0.019	0.522	-0.032	0.393	-0.027	0.575	0.720
uPA	0.023	0.408	0.013	0.738	0.011	0.799	0.753
VEGF-A	-0.007	0.808	-0.004	0.919	-0.004	0.933	0.854

Model 3 was adjusted for age, sex, study cohort, BMI, HbA1c, diabetes duration, total cholesterol, triglycerides, use of lipid-lowering drugs, use of NSAIDs, use of

antithrombotic medication, use of antidepressant medication and the number of diabetes-related comorbidities.

T1D, type 1 diabetes; T2D, type 2 diabetes; Total, people with type 1 or 2 diabetes.

See **Supplementary Table 2** for a list of full biomarker names.

Supplementary Table 10. Associations of biomarkers of inflammation and anhedonia symptoms in the CES-D score (model 3)

Biomarkers	Total		T1D		T2D		$P_{interaction}$
	β	*P*	β	*P*	β	*P*	
ADA	0.001	0.961	-0.006	0.861	0.017	0.712	0.515
Axin-1	0.020	0.456	-0.030	0.418	0.079	0.059	0.067
Caspase-8	0.018	0.527	-0.008	0.830	0.046	0.286	0.326
CCL4	-0.020	0.484	-0.049	0.171	0.026	0.563	0.087
CCL19	-0.007	0.809	-0.033	0.384	0.028	0.521	0.166
CCL20	-0.007	0.810	-0.005	0.898	-0.013	0.741	0.998
CCL23	0.011	0.677	-0.002	0.950	0.038	0.378	0.508
CCL25	0.048	0.088	0.047	0.190	0.055	0.236	0.849
CCL28	0.001	0.971	-0.003	0.925	0.010	0.848	0.996
CD5	0.052	0.066	0.032	0.408	**0.087**	**0.039**	0.167
CD6	0.001	0.960	-0.034	0.340	0.058	0.187	**0.041**
CD8A	0.054	0.054	0.022	0.546	**0.107**	**0.018**	0.076
CD40	0.018	0.535	-0.004	0.911	0.056	0.202	0.328
CD244	-0.010	0.723	-0.056	0.089	0.096	0.050	**0.006**
CDCP1	0.040	0.214	0.018	0.694	0.052	0.286	0.515
CSF-1	0.0001	0.999	-0.022	0.561	0.039	0.424	0.117
CST5	-0.007	0.808	-0.0001	0.999	-0.025	0.579	0.540
CX3CL1	-0.006	0.827	-0.040	0.274	0.036	0.420	0.228
CXCL1	0.040	0.156	0.023	0.536	0.062	0.153	0.274
CXCL5	0.008	0.793	0.006	0.882	0.0001	0.997	0.666
CXCL6	0.042	0.131	0.001	0.968	**0.106**	**0.017**	**0.028**
CXCL9	0.007	0.805	0.037	0.313	-0.025	0.596	0.153
CXCL10	-0.012	0.656	-0.004	0.921	-0.028	0.503	0.638
CXCL11	0.022	0.418	0.029	0.400	0.015	0.747	0.845
DNER	-0.012	0.681	-0.037	0.365	-0.006	0.893	0.738
EIF4EBP1	0.038	0.185	0.038	0.293	0.033	0.490	0.960
EN-RAGE	0.013	0.632	-0.012	0.739	0.043	0.321	0.273
Eotaxin	0.056	0.060	**0.092**	**0.017**	-0.022	0.663	0.053
FGF-5	-0.025	0.377	0.007	0.834	-0.093	0.056	0.064
FGF-19	0.0003	0.991	-0.028	0.463	0.035	0.435	0.369
FGF-21	0.038	0.265	0.041	0.336	0.066	0.287	0.439
Flt3L	0.015	0.596	0.029	0.450	-0.022	0.642	0.395
GDNF	0.044	0.112	-0.004	0.901	**0.132**	**0.005**	0.059
HGF	0.048	0.097	0.048	0.200	0.045	0.346	0.598
IFNγ	-0.005	0.862	0.053	0.157	-0.069	0.096	**0.026**
IL-2RB	0.021	0.449	0.006	0.856	0.049	0.275	0.555
IL-6	0.023	0.455	0.017	0.676	0.028	0.579	0.560

IL-7	-0.009	0.757	-0.009	0.813	-0.004	0.920	0.761
IL-8	**0.067**	**0.020**	0.033	0.430	**0.092**	**0.023**	0.333
IL-10	0.040	0.139	0.057	0.148	0.034	0.371	0.728
IL-10RA	-0.040	0.143	-0.034	0.310	-0.025	0.584	0.713
IL-10RB	0.041	0.153	0.001	0.986	**0.115**	**0.013**	**0.031**
IL-12B	0.007	0.820	-0.028	0.477	0.070	0.117	0.091
IL-15RA	0.013	0.639	-0.013	0.736	0.056	0.200	0.261
IL-17A	0.035	0.193	0.024	0.533	0.046	0.235	0.619
IL-17C	-0.030	0.274	-0.030	0.473	-0.026	0.489	0.898
IL-18	0.045	0.116	0.051	0.161	0.042	0.357	0.925
IL-18R1	0.046	0.115	-0.039	0.331	**0.153**	**0.001**	**<0.001**
LAP TGFβ1	-0.008	0.761	-0.034	0.313	0.033	0.474	0.156
LIF-R	0.022	0.424	-0.055	0.124	**0.142**	**0.002**	**0.002**
MCP-1	0.016	0.564	0.0001	0.997	0.028	0.545	0.501
MCP-2	-0.039	0.152	-0.061	0.090	-0.002	0.957	0.182
MCP-3	0.043	0.173	0.076	0.079	-0.010	0.834	0.467
MCP-4	-0.012	0.664	-0.053	0.144	0.039	0.398	0.092
MIP-1α	0.044	0.124	0.015	0.688	**0.094**	**0.040**	0.094
MMP-1	0.029	0.285	0.047	0.197	0.0003	0.995	0.506
MMP-10	**0.055**	**0.048**	**0.072**	**0.038**	0.031	0.526	0.323
NT-3	-0.048	0.091	**-0.090**	**0.011**	0.025	0.610	**0.031**
OPG	-0.008	0.791	-0.075	0.069	0.058	0.194	**0.040**
OSM	0.038	0.174	0.051	0.155	0.020	0.650	0.926
PD-L1	-0.007	0.794	-0.006	0.870	-0.006	0.879	0.926
SCF	-0.043	0.148	-0.044	0.319	-0.050	0.216	0.560
SIRT2	0.017	0.533	-0.030	0.382	**0.096**	**0.033**	**0.019**
SLAMF1	0.030	0.297	0.032	0.424	0.022	0.584	0.927
ST1A1	0.0004	0.988	-0.015	0.661	0.034	0.468	0.473
STAMBP	0.023	0.404	-0.013	0.708	0.079	0.070	0.086
TGFα	0.046	0.095	0.051	0.159	0.039	0.361	0.751
TNFRSF9	0.054	0.056	0.055	0.172	0.060	0.146	0.986
TNFSF14	0.038	0.162	0.034	0.328	0.045	0.313	0.469
TNFα	0.010	0.712	0.013	0.721	0.011	0.802	0.789
TNFβ	-0.011	0.697	**-0.092**	**0.023**	0.052	0.191	**0.007**
TRAIL	-0.017	0.544	0.016	0.664	-0.073	0.118	0.221
TRANCE	-0.013	0.659	0.004	0.909	-0.033	0.487	0.802
TWEAK	-0.027	0.348	**-0.082**	**0.030**	0.039	0.400	**0.040**
uPA	0.024	0.379	-0.019	0.608	0.056	0.197	0.250
VEGF-A	0.007	0.790	0.031	0.384	-0.029	0.541	0.498

Model 3 was adjusted for age, sex, study cohort, BMI, HbA1c, diabetes duration, total cholesterol, triglycerides, use of lipid-lowering drugs, use of NSAIDs, use of

antithrombotic medication, use of antidepressant medication and the number of diabetes-related comorbidities.

T1D, type 1 diabetes; T2D, type 2 diabetes; Total, people with type 1 or 2 diabetes.

See **Supplementary Table 2** for a list of full biomarker names.

Supplementary Fig. 1. Study population

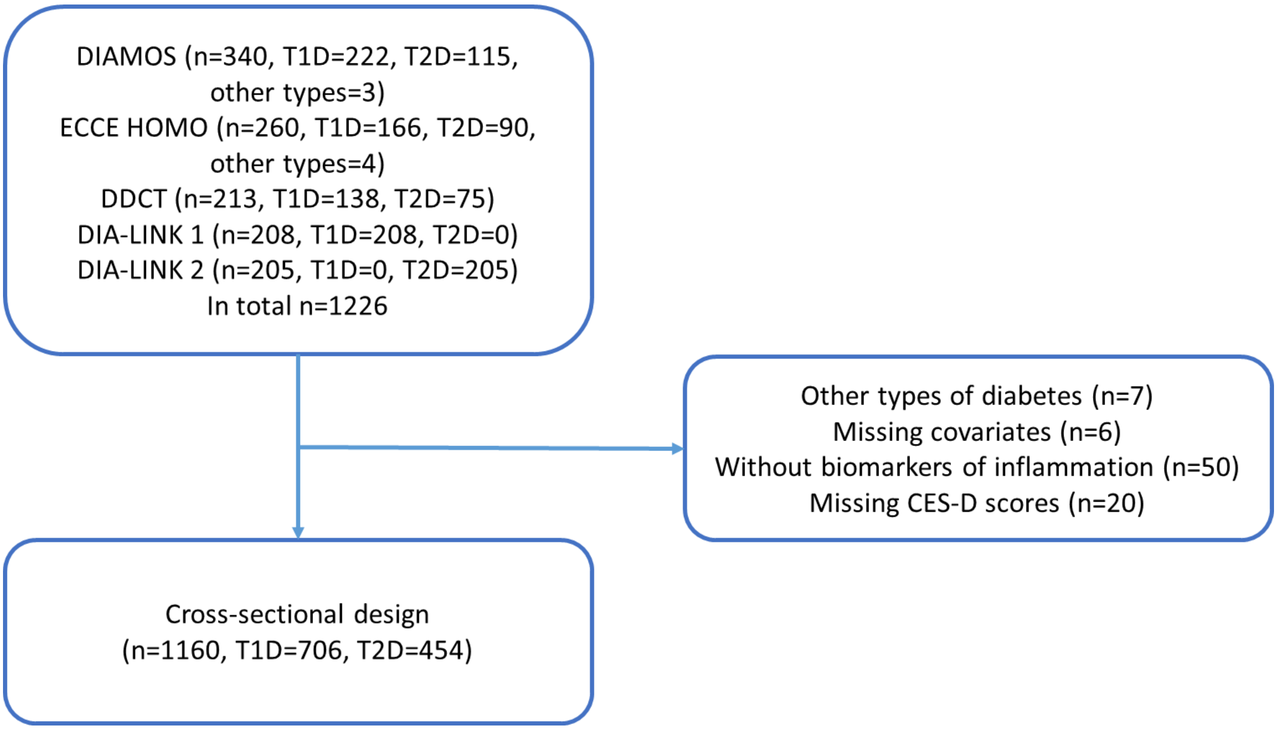

CES-D, Center for Epidemiological Studies-Depression; DDCT, Depression and Diabetes Control Trial; DIA-LINK 1/2, Towards a Better Understanding of Diabetes Distress, Depression and Poor Glycaemic Control in T1D/T2D; DIAMOS, Diabetes Motivation Strengthening; ECCE HOMO, Evaluation of a Stepped Care Approach to Manage Depression in Diabetes; NSAIDs, non-steroidal anti-inflammatory drugs; PAOD, peripheral arterial occlusive disease; T1D, type 1 diabetes; T2D, type 2 diabetes.

Supplementary Fig. 2. Correlation matrix of 76 biomarkers of inflammation

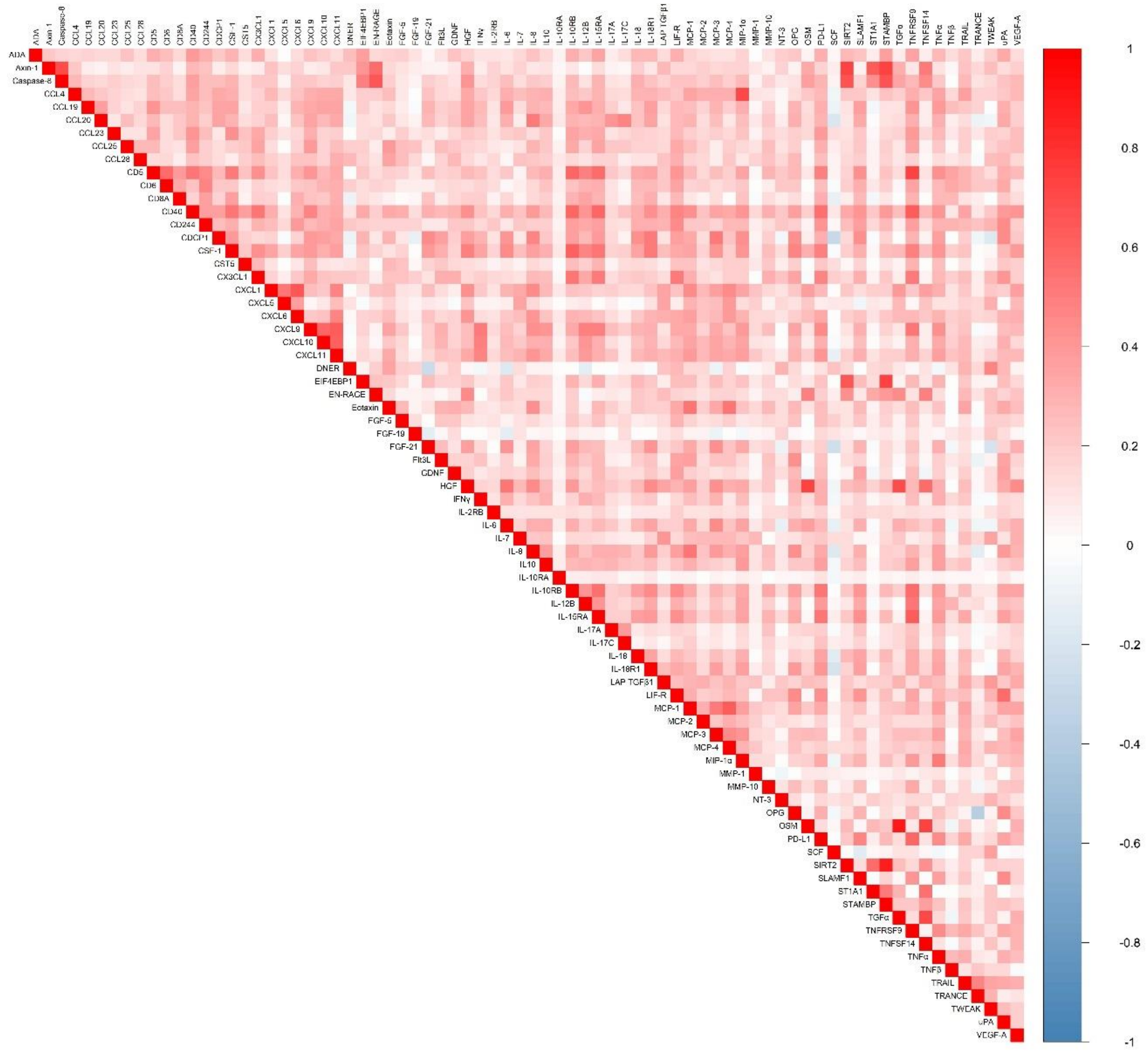

The matrix shows Pearson's correlation coefficients (r) for pairwise correlations between biomarkers of inflammation in the total study sample.

See **Supplementary Table 2** for a list of full biomarker names.

Supplementary Fig. 3. Chord diagram of correlations between 76 biomarkers of inflammation and participant characteristics

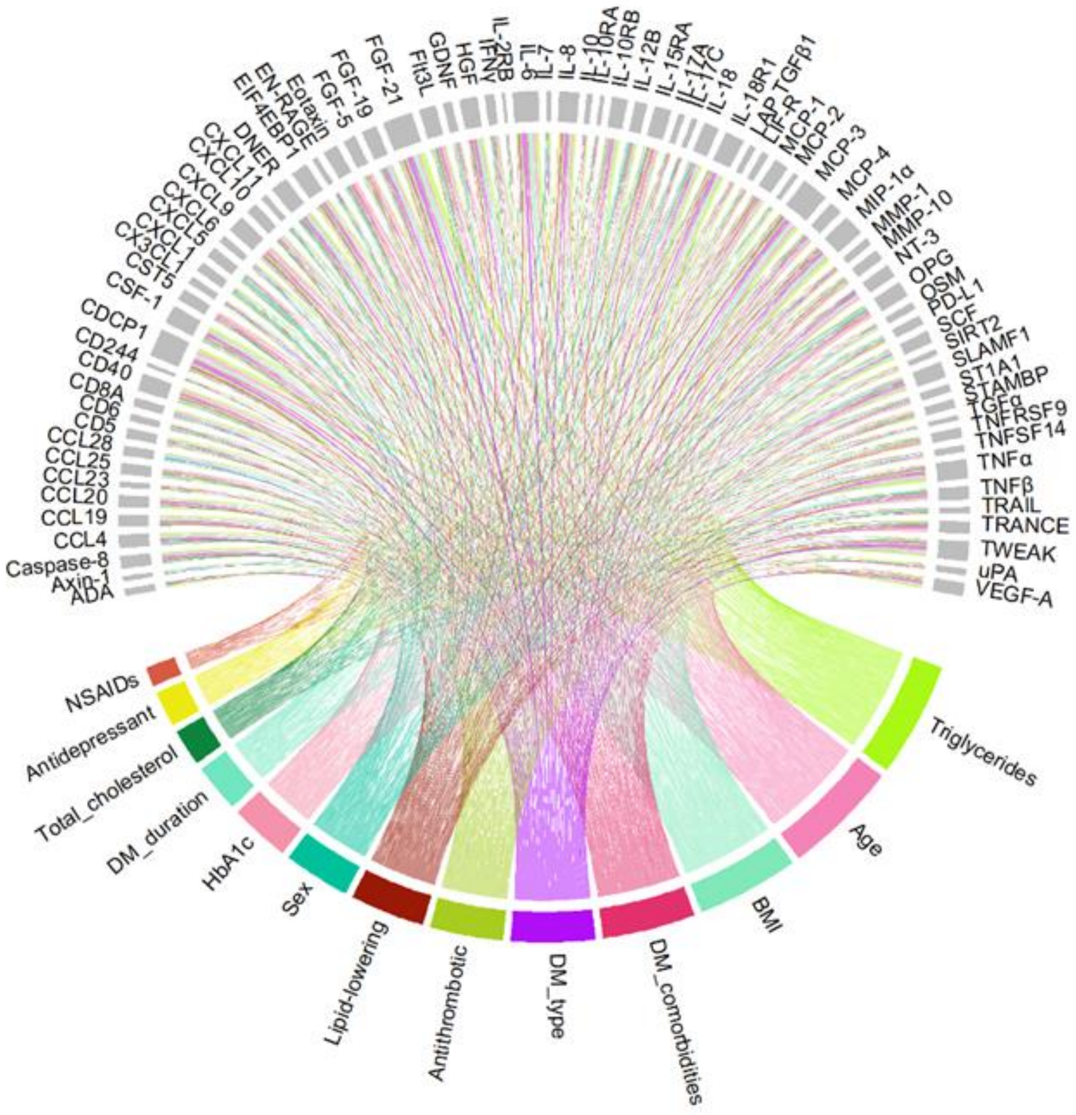

The diagram indicates the number of pairwise correlations between biomarkers of inflammation and participant characteristics.

BMI, body mass index; DM, diabetes mellitus; NSAIDs: non-steroidal anti-inflammatory drugs.

See **Supplementary Table 2** for a list of full biomarker names.

Supplementary Fig. 4 Heat map of correlations between 76 biomarkers of inflammation and participant characteristics

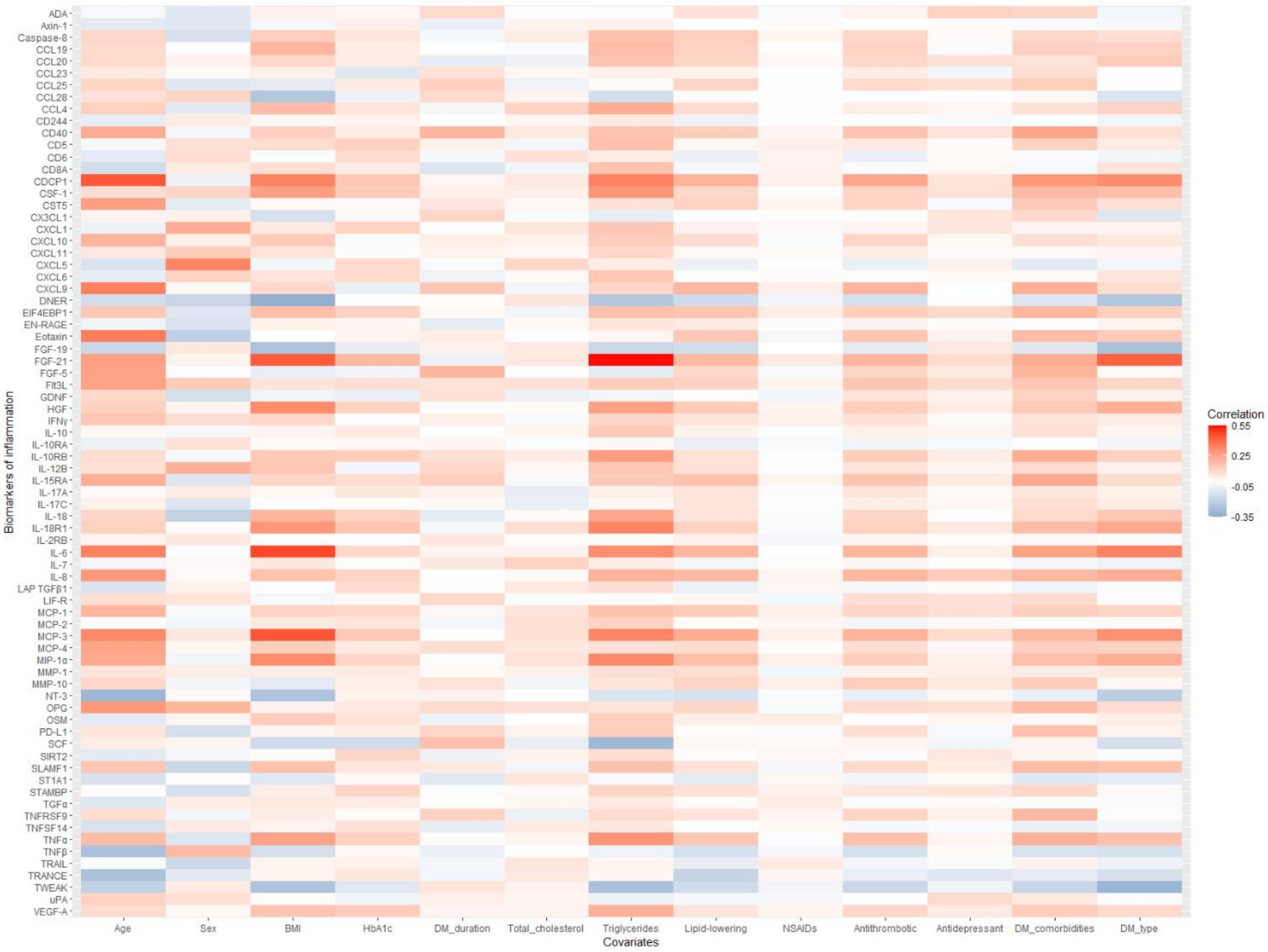

The diagram visualises pairwiese correlations between biomarkers of inflammation and participant characteristics. For the correlation analysis, categorical variables were labelled as follows: sex, 1=male, 2=female; use of lipid-lowering drugs, NSAIDs, antithrombotic drugs or antidepressant drugs: 0=no, 1=yes; DM_type, 1=type 1 diabetes, 2=type 2 diabetes. All others variables were used as continuous variables.

BMI, body mass index; DM, diabetes mellitus; NSAIDs: non-steroidal anti-inflammatory drugs; T1D, type 1 diabetes; T2D, type 2 diabetes.

See **Supplementary Table 2** for a list of full biomarker names.

Supplementary Fig. 5. Heat map summarising associations of biomarkers of inflammation with the CES-D total score

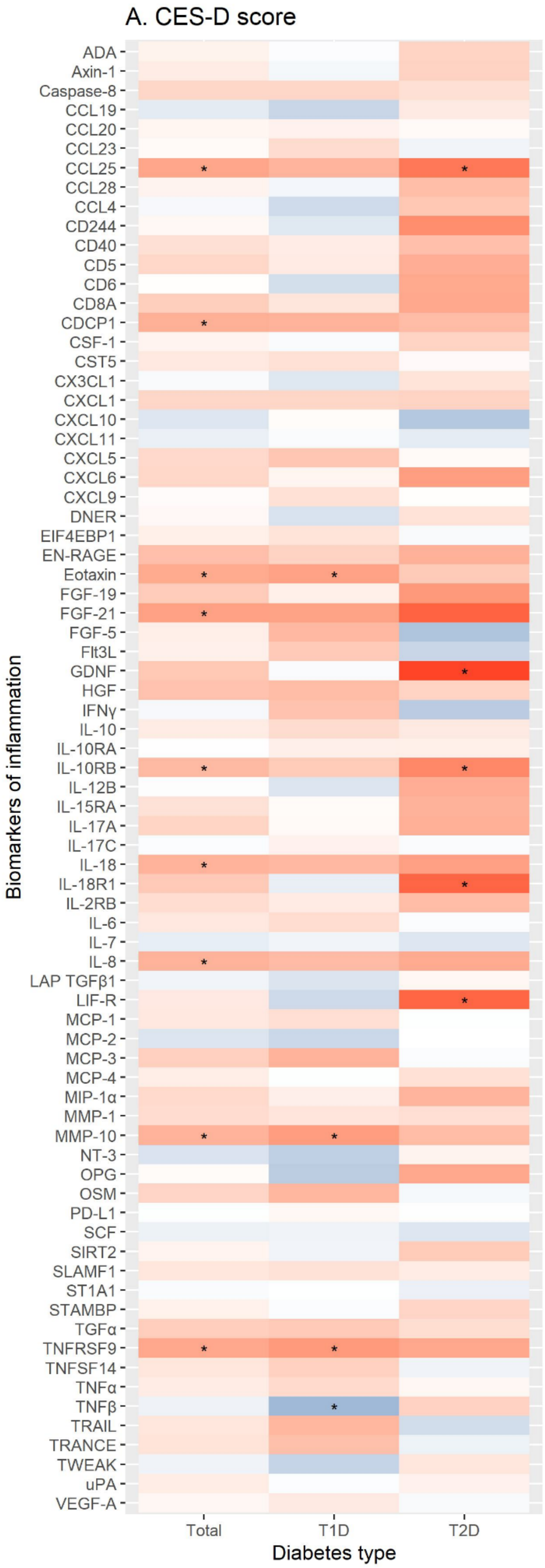

Supplementary Fig. 6. Heat map summarising associations of biomarkers of inflammation with cognitive-affective symptoms

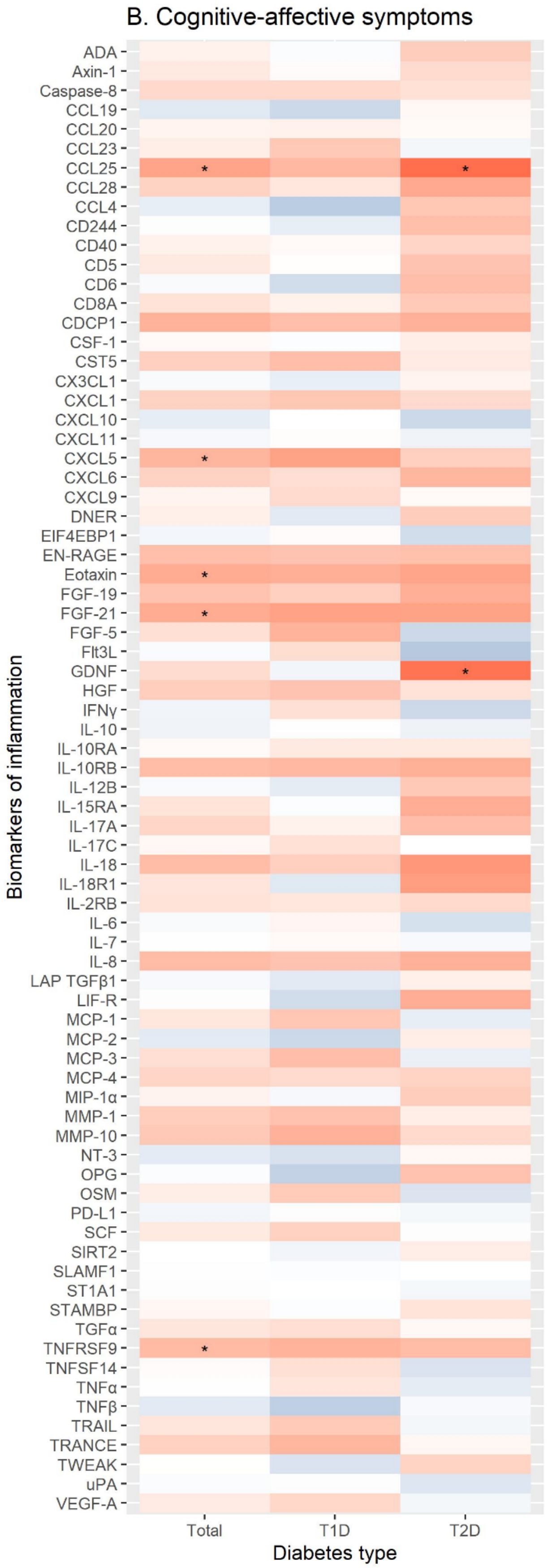

Supplementary Fig. 7. Heat map summarising associations of biomarkers of inflammation with somatic symptoms

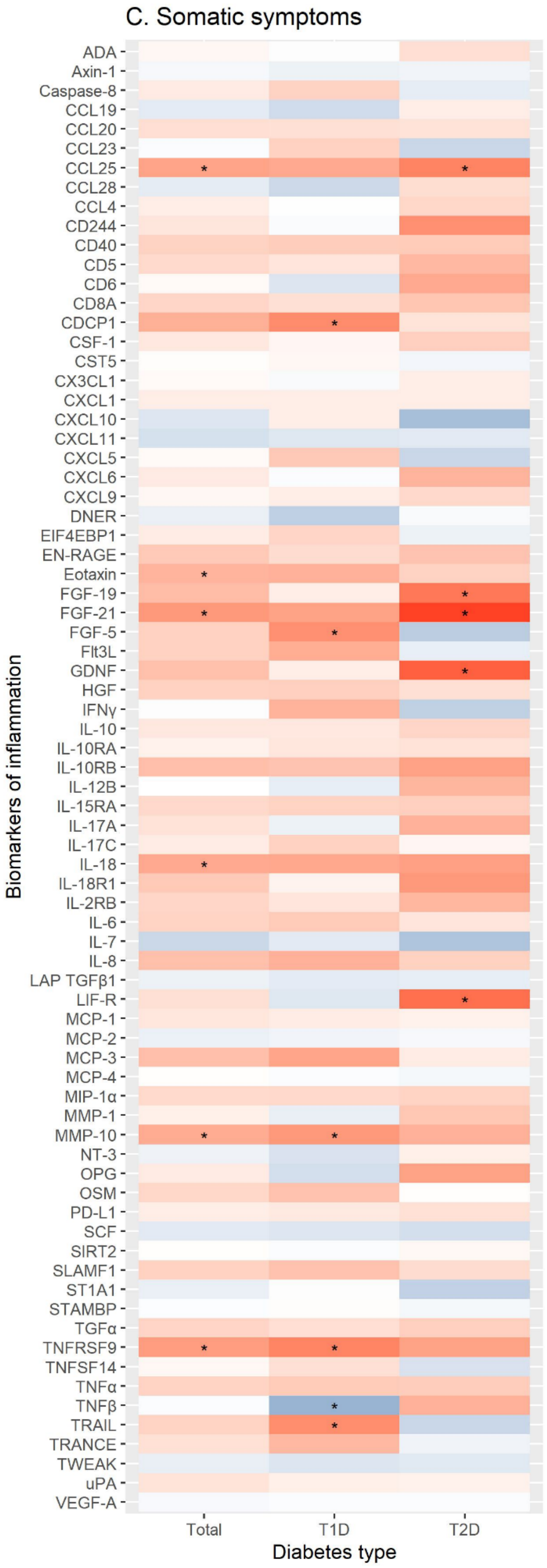

Supplementary Fig. 8. Heat map summarising associations of biomarkers of inflammation with anhedonia symptoms

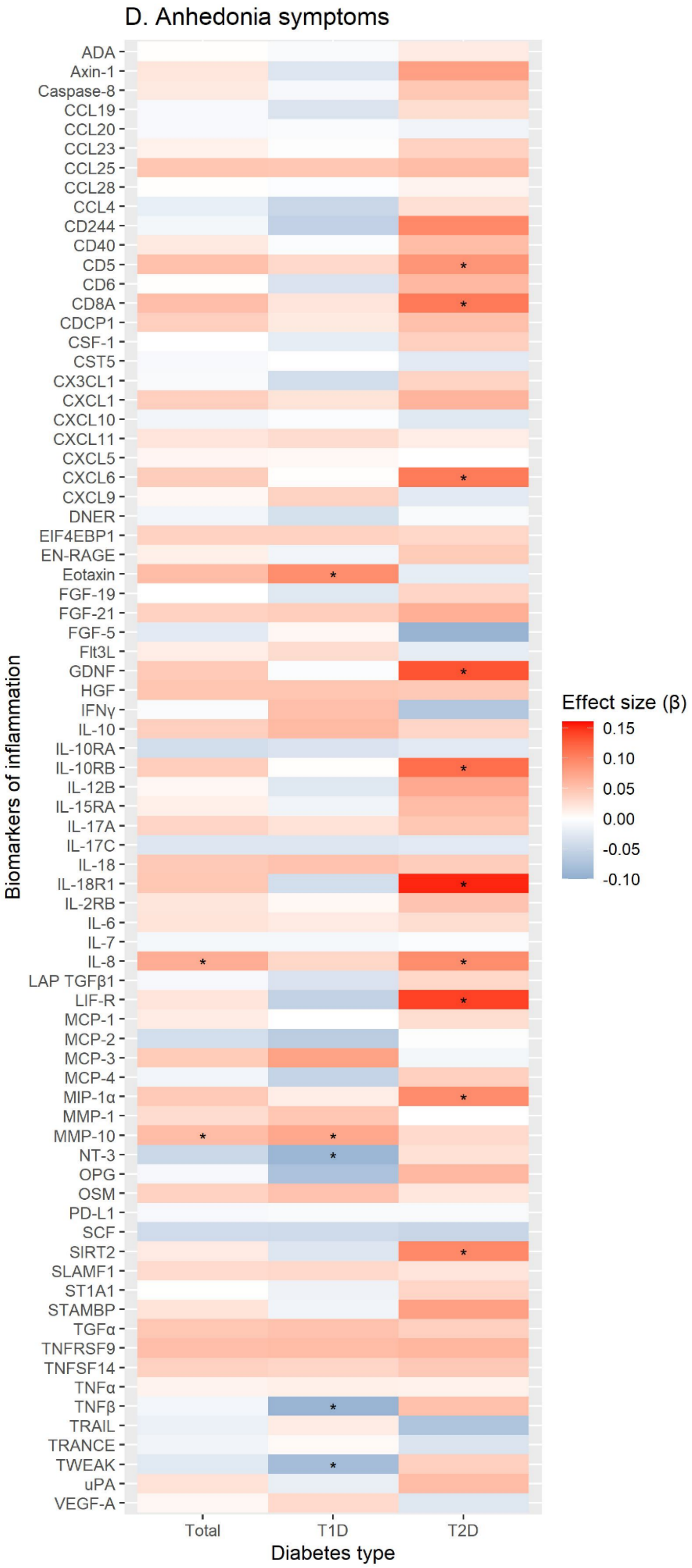

Supplement: Supplementary file 1 — Supplemental tables and figures [file 41398_2024_3209_MOESM1_ESM.pdf]
